# Characterization and evaluation of gene fusions as a measure of genetic instability and disease prognosis in prostate cancer

**DOI:** 10.1186/s12885-023-11019-6

**Published:** 2023-06-22

**Authors:** Carolin Schimmelpfennig, Michael Rade, Susanne Füssel, Dennis Löffler, Conny Blumert, Catharina Bertram, Angelika Borkowetz, Dominik J. Otto, Sven-Holger Puppel, Pia Hönscheid, Ulrich Sommer, Gustavo B. Baretton, Ulrike Köhl, Manfred Wirth, Christian Thomas, Friedemann Horn, Markus Kreuz, Kristin Reiche

**Affiliations:** 1 Department of Diagnostics, Fraunhofer Institute for Cell Therapy and Immunology, Leipzig, Germany; 2 Department of Urology, University Hospital and Faculty of Medicine, Technische Universität Dresden, Dresden, Germany; 3 Institute of Pathology, University Hospital and Faculty of Medicine, Technische Universität Dresden, Dresden, Germany; 4 Institute of Clinical Immunology, Medical Faculty, University Hospital, University of Leipzig, Leipzig, Germany

**Keywords:** Biomarker, Gene fusion, Genomic instability, Molecular diagnostic testing, Molecular pathology, Next-generation sequencing, Prognosis, Prostate cancer, Transcriptome

## Abstract

**Background:**

Prostate cancer (PCa) is one of the most prevalent cancers worldwide. The clinical manifestations and molecular characteristics of PCa are highly variable. Aggressive types require radical treatment, whereas indolent ones may be suitable for active surveillance or organ-preserving focal therapies. Patient stratification by clinical or pathological risk categories still lacks sufficient precision. Incorporating molecular biomarkers, such as transcriptome-wide expression signatures, improves patient stratification but so far excludes chromosomal rearrangements. In this study, we investigated gene fusions in PCa, characterized potential novel candidates, and explored their role as prognostic markers for PCa progression.

**Methods:**

We analyzed 630 patients in four cohorts with varying traits regarding sequencing protocols, sample conservation, and PCa risk group. The datasets included transcriptome-wide expression and matched clinical follow-up data to detect and characterize gene fusions in PCa. With the fusion calling software Arriba, we computationally predicted gene fusions. Following detection, we annotated the gene fusions using published databases for gene fusions in cancer. To relate the occurrence of gene fusions to Gleason Grading Groups and disease prognosis, we performed survival analyses using the Kaplan–Meier estimator, log-rank test, and Cox regression.

**Results:**

Our analyses identified two potential novel gene fusions, *MBTTPS2,L0XNC01::SMS* and *AMACR::AMACR*. These fusions were detected in all four studied cohorts, providing compelling evidence for the validity of these fusions and their relevance in PCa. We also found that the number of gene fusions detected in a patient sample was significantly associated with the time to biochemical recurrence in two of the four cohorts (log-rank test, *p*-value < 0.05 for both cohorts). This was also confirmed after adjusting the prognostic model for Gleason Grading Groups (Cox regression, *p*-values < 0.05).

**Conclusions:**

Our gene fusion characterization workflow revealed two potential novel fusions specific for PCa. We found evidence that the number of gene fusions was associated with the prognosis of PCa. However, as the quantitative correlations were only moderately strong, further validation and assessment of clinical value is required before potential application.

**Supplementary Information:**

The online version contains supplementary material available at 10.1186/s12885-023-11019-6.

## Background

Prostate cancer (PCa) is the second most common malignant disease in men and the fifth leading cause of cancer-related death worldwide [1]. Localized PCa shows a broad spectrum of clinical behaviors, ranging from indolent to aggressive forms, with varying genotypes and phenotypes [2]. Aggressive forms of PCa are commonly treated with radical prostatectomy (RP) or radiotherapy [3]. In contrast, localized, low-risk, and clinically non-significant PCa that displays no evident risk of metastasis is often actively monitored. Risk categories relying solely on clinical parameters lack sufficient precision [4]. Thus, understanding the molecular differences of PCa and their implications, as well as their suitability as additional biomarkers for PCa prognosis, is urgently needed.

In the past, PCa prognosis has benefitted from a detailed understanding of the relationship between the clinical course of the disease and patient-specific molecular profiles. Several studies have used extended statistical models that combine clinical factors like Gleason Grading Groups (GGG) or prostate-specific antigen (PSA) levels with multiparametric imaging, genomic or transcriptomic markers, or other biochemical markers to measure the risk of tumor progression [5–7]. However, none of the prognostic models incorporates parameters reflecting the status of chromosomal rearrangements and transcriptional disorganization in a patient sample, even though gene fusions are known to drive PCa development and progression [8].

Gene fusions are chimeric genes that combine at least two parental genes. Such chromosomal rearrangements are critical in tumorigenesis by introducing new or altered chimeric proteins as well as non-coding RNAs (ncRNAs) to the cell, altering the regulation of cellular pathways, and thus supporting the evolution of cancer cells [2, 9, 10]. To date, more than ten thousand PCa-associated gene fusions with variable occurrences have been identified [11, 12]. PCa is a tumor with low mutational burden but large genomic intra- and inter-patient heterogeneity; thus, patients carry a variety of gene fusion combinations [13–15]. In contrast to most other solid tumors, gene fusions in PCa are a central element of tumorigenesis [2, 6]. In particular, gene fusions formed at the transcriptional level are associated with PCa development and progression [16]. One of the most prominent gene fusions in PCa is *TMPRSS2::ERG.* It occurs in approximately 50% of PCa patients of European descent, as well as in about 25% of patients of Asian and African descent [17, 18]. *TMPRSS2::ERG* is known to influence multiple cellular functions, such as cell invasion, metastasis, and the differentiation of prostate epithelium upon constitutive *ERG* overexpression [19]. The altered expression of *ERG* is known to induce changes in many different cellular pathways, such as the PI3K or Wnt signaling pathways, making *ERG* a crucial element in PCa development and progression [18].

In this study, we characterized known and novel gene fusions, including fusions of ncRNAs, by analyzing four RNA sequencing cohorts. We investigated whether the detected gene fusions are potentially suitable molecular markers for PCa risk stratification. We also evaluated the occurrence of single gene fusions in two newly sequenced PCa cohorts with long-term follow-up (*n* = 40 and *n* = 176 patient samples), The Cancer Genome Atlas (TCGA) prostate adenocarcinoma (PRAD) cohort (*n* = 332 patient samples), and a set of early-onset PCa samples from Gerhauser et al. (*n* = 82 patient samples) [20]. This allowed us to assess the familiarity and functionality of detected gene fusions and associated their detection with clinical outcome, taking into account known clinical factors such as Gleason Grading Group and prognostic gene expression. Our hypothesis was that the overall number of gene fusions in a PCa patient sample might serve as a surrogate marker for the degree of genomic dysregulation and therefore be associated with disease progression. Thus, we aimed to improve strategies for stratifying PCa patients in early clinical decision-making.

## Methods

### Cohort description

**Fig. 1:**
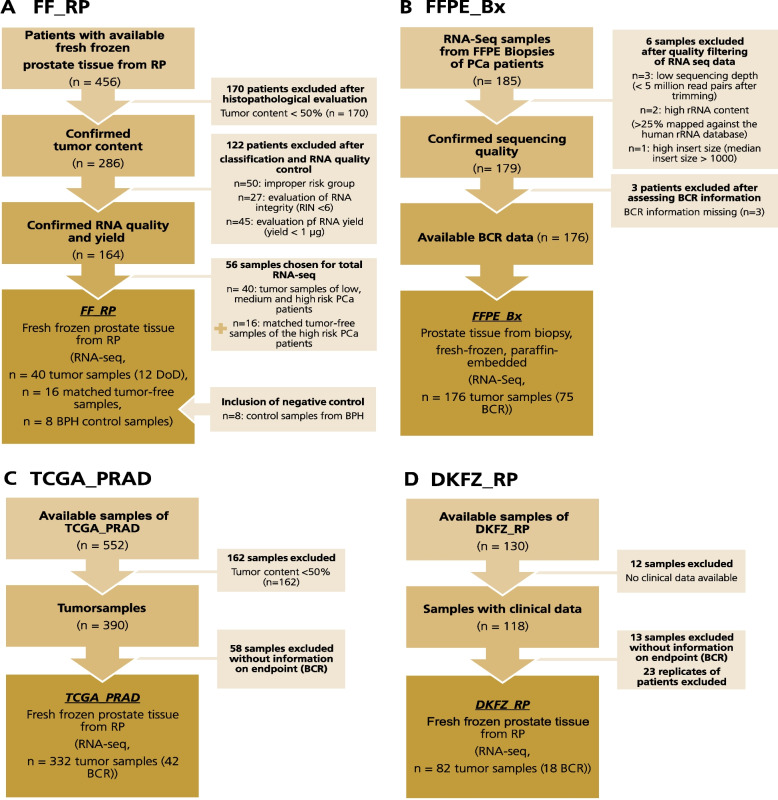
Overview of the cohorts included in this study. The flowcharts depict the number of patients included in each cohort as well as exclusion criteria according to REMARK [25]. **A** FF_RP: 64 samples of the fresh-frozen tissue specimens fulfilled all inclusion criteria. 40 of those samples were tumor samples, and 16 were matched tumor-free samples. The remaining eight samples were tissue specimens from patients with benign prostatic hyperplasia and served as controls. **B** FFPE_Bx: 176 patients fulfilled all inclusion criteria. All samples were derived from FFPE biopsies. **C** TCGA_PRAD: Of the initial 552 samples, we included 332 samples that met our inclusion criteria. **D** DKFZ_RP: We downloaded the data for 130 samples from the EGA archive. Of those, we included 82 samples in our analyses. Patient characteristics of the cohorts are shown in Tables S1 and S2 (Additional file 1). BCR: biochemical recurrence; Bx: biopsy; DoD: Death of Disease; FF: fresh-frozen; FFPE: formalin-fixed paraffin-embedded; RP: radical prostatectomy; RIN:RNA integrity number

We assessed primary PCa tissue specimens with follow-up data from four different cohorts. The first cohort, FF_RP, consisted of 40 fresh-frozen tissue specimens from 456 PCa patients who underwent radical prostatectomy (RP) between 1995 and 2008 at the Department of Urology of the University Hospital Dresden (Germany) (Fig. 1A). None of the patients had received neoadjuvant therapy prior to surgery. Kreuz et al. [6] have provided a comprehensive description of the clinical characterization of the cohort and information on the processing of the 456 tumor samples. After stringent quality control, 164 tissue specimens with high tumor cell content (TCC) and sufficient RNA quality and yield remained (Fig. 1A). From these 164 samples, we selected the tissue specimens of 40 PCa patients for deep sequencing (~ 200 million reads). All remaining samples were analyzed with microarrays (Agilent)—see Kreuz et al. [6]. The patient samples were divided into eight clinical risk groups (Table S1 and Kreuz et al. [6]) based on Gleason Score (GS), the presence of regional lymph node metastases (pN), and the occurrence of death of disease (DoD), which was the primary endpoint of this cohort. For our analyses, we have enriched certain risk groups with patient samples with observed events (see Kreuz et al. [6] for details). In addition to the tumor samples, we included tissue specimens from eight benign prostate hyperplasia (BPH) patients and 16 matched tumor-free tissue specimens (TCC = 0–5%) from high-risk patients (Fig. 1A, Table S1). These samples served as control samples for the computational prediction of gene fusions. We performed RNA sequencing of the tumor-free and control samples similar to the process for the tumor samples described by Kreuz et al. [6]. All samples were sequenced relatively deep, with approximately 200 million reads per sample, allowing the detection of rare transcripts. To validate gene expression, the other specimens were analyzed using custom expression arrays (Agilent), as described by Kreuz et al. [6].

As a second cohort, we included 185 formalin-fixed and paraffin-embedded (FFPE) samples from biopsies collected at the Department of Urology, University Hospital Dresden (Germany), between 2007 and 2013 (FFPE_Bx, Fig. 1B). All patients in this cohort were treated with RP and did not receive neoadjuvant therapy prior to surgery. After exclusion of six samples with insufficient quality and three patients without biochemical recurrence (BCR) data, 176 samples remained. As described by Rade et al. [21], we chose a total RNA sequencing protocol.

For both cohorts, FF_RP and FFPE_Bx, the Internal Review Board at the Technische Universität Dresden (EK194092004, EK195092004, EK59032007) approved the study, and all patients provided written informed consent. Routine histopathological examination of the surgical specimens revealed clinicopathological parameters. Serum prostate-specific antigen (PSA) levels were determined preoperatively. Information on disease progression, patients' survival, and cause of death was obtained from the patient's medical records, from the treating urologists or general practitioners, or from records of the regional tumor registry.

As a third cohort, we used poly(A)-enriched transcriptome-wide expression data from 332 patients from the TCGA_PRAD cohort from TCGA (Fig. 1C) [12]. The inclusion of this thoroughly studied dataset, in our analyses supported the results in our own cohorts and validated our workflow by allowing comparison between our results and the published results for the cohort. This paper's Table S2 as well as the TCGA publications by Abeshouse et al. [12] and Kreuz et al. [6] provide comprehensive descriptions of the data source.

As a fourth cohort, we included a cohort of patients with early-onset PCa from Gerhauser et al. [20], DKFZ_RP (Fig. 1D). It provides poly(A)-RNA-sequencing data of 82 fresh-frozen samples with matched information on clinical variables such as GS and BCR. Some patients (*n* = 6) in this cohort were represented by multiple samples. After confirming the consistency of the *TMPRSS2::ERG* fusion for every replicate, we reduced the number of samples per patient to one (the first available sample) (Table S13C). With its fresh-frozen samples, the cohort provided well-conserved RNA, whereas the nature of the early-onset PCa samples implied a molecular difference [20, 22, 23] from the samples previously included in this study.

Each cohort provided unique combinations of characteristics. While FF_RP, TCGA_PRAD, and DKFZ_RP consisted of fresh-frozen tissue specimens from radical prostatectomy, FFPE_Bx specimens were formalin-fixed and paraffin-embedded biopsy samples. This specimen and conservation type is most commonly used in clinical practice. In addition, the cohorts differed in RNA-sequencing protocols, with total RNA sequencing for FF_RP and FFPE_Bx versus poly(A)-enriched RNA libraries for TCGA_PRAD and DKFZ_RP. Total RNA sequencing enables analysis of all ncRNA families, whereas poly(A)-sequencing captures only RNA with a poly(A) tail, such as mRNA and some long ncRNA families [24]. The DKFZ_RP cohort also allowed us to characterize fusions from the perspective of early-onset PCa and to assess the prognostic potential of gene fusions in an early-onset PCa cohort.

The primary endpoints were DoD for FF_RP and BCR for TCGA_PRAD, DKFZ_RP, and FFPE_Bx. Patients who did not experience an event or those who dropped out for other reasons were censored at the last follow-up. Table S2 in Additional File 1 provides further information on the technical and clinicopathological characteristics of all the cohorts.

### Preprocessing of Transcriptome-wide Data

For FF_RP and FFPE_Bx, we processed the total RNA sequencing data with demultiplexing, adapter-clipping, and quality control, as described in the supplementary material by Kreuz et al. [6]. The sequencing depth of the cohorts per sample was approximately 200 million reads (FF_RP) and 50 million reads (FFPE_Bx). Kreuz et al. [6] described the processing of the TCGA_PRAD poly(A)-enriched sequencing data and associated clinical data in the supplementary material. We estimated the sequencing depth via the mean of the raw read counts to be approximately 56 million reads per sample. The poly(A)-enriched sequencing data of the TCGA_PRAD cohort are provided as BAM files aligned to the human reference genome GRCh38/hg38 using the STAR aligner. To retrieve the data formats accessible by Arriba, we applied Picard Tools version 1.118 (http://broadinstitute.github.io/picard/) to revert the supplied BAM files to the FASTQ format with SamToFastq. According to Rade et al.'s [21] description, the DKFZ_RP poly(A)-enriched sequencing data was downloaded in FASTQ format using pyEGA3 software [26] as well as the relevant clinical data from cBioportal. We excluded patients from our study without matched clinical data or complete information on BCR and limited samples to one replicate per patient.

### Detection of gene fusions

The FASTQ files of all samples served as inputs for gene fusion detection with Arriba (version 1.2.0) [27], using the STAR aligner (version 2.6.1c) [28] to map reads to the human genome version hg38. Using the workflow manager Nextflow (version 19.10.0) with an adapted script of the Arriba pipeline (version 1) assured parallel and reproducible data processing. To increase the sensitivity of Arriba, we combined a list of known gene fusions provided as a whitelist by Arriba with a list of ETS-specific fusion partners provided by Tandefelt et al. [29] (Table S3). With including a list of known gene fusions, we followed the same strategy as the TCGA consortium in its PCa publication in 2015 [11, 12]. For all other Arriba and STAR options, we used default values, as provided in the Arriba manual [27]. This also included the Arriba blacklist option, for which the software provided a list of recurrent alignment artifacts and gene fusions present in healthy tissue.

### Quality assessment of gene fusions

We merged the Arriba results for all individual tissue specimens (< sample > .fusions.tsv files) into a single file for each cohort. These files contain breakpoints from the fusions, the number of reads supporting a specific gene fusion, and information on filters applied to specific fusions. Arriba also assigned a confidence level to each predicted fusion based on the technical and biological aspects of the fusion. To assess the quality of the fusion-calling results, we determined the number of fusions per cohort (designated as *the number of fusions* in the following text), per sample, and per confidence level. When multiple identical gene fusions were detected in individual samples, we selected the fusion with the highest confidence and highest number of supporting reads for further analysis. To assess the unique fusions in a cohort, each detected fusion was counted once per cohort. For all steps, we used the statistical computing environment R 4.0.0 [30]. To validate our pipeline, we used gene fusions covering the genes *ERG*, *ETV1*, *ETV4*, and *FLI1* reported by the TCGA consortium for the TCGA_PRAD cohort as a reference. The annotated data from the TCGA consortium was provided by the cBioPortal for Cancer Genomics database [31] in the Prostate Adenocarcinoma study (TCGA, Cell 2015). We compared the samples carrying an *ERG*-fusion as published by the TCGA consortium with those for which we detected an *ERG*-fusion in TCGA_PRAD with the Arriba pipeline one-to-one and created contingency tables of the results. The same analyses were performed for *ETV1*, *ETV4*, and *FLI1*. To further evaluate the detection of fusions in FF_RP, FFPE_Bx, and DKFZ_RP, we examined which of the above mentioned genes were found in fusions in these three cohorts and compared their frequency of occurrence with the TCGA_PRAD cohort.

Based on the confidence levels Arriba determined, we filtered candidate gene fusions for our analyses. If not otherwise mentioned, we performed the following analyses with high-confidence gene fusions. To visualize high-confidence gene fusions per sample, we used the R package ggplot2 (version 3.3.6) [32].

### Characterization and annotation of detected gene fusions

To characterize gene fusion, we applied different criteria depending on the properties of the cohorts. Fresh-frozen tissue specimen cohorts (FF_RP, *n* = 40; TCGA_PRAD, *n* = 332) were used as discovery cohorts for potential novel gene fusions. Gene fusions that Arriba [27] classified as "high confidence" and found in at least one discovery cohort we considered as fusions of interest. In contrast to fresh-frozen tissue samples, the quality of RNA isolated from FFPE biopsies decreases with sample age, resulting in lower accuracy in detecting gene fusions [33]. However, FFPE biopsies correspond to routine clinical samples and are thus helpful in assessing gene fusions as close as possible to clinical conditions. To counteract the lower accuracy in detecting gene fusions in FFPE samples, we matched gene fusions that were detected in at least one discovery cohort (at high confidence) with gene fusions detected in FFPE_Bx and added them regardless of their confidence level in FFPE_Bx. This set of fusions was combined with all remaining gene fusions detected with high confidence in FFPE_Bx (Fig. S1). We annotated the discovered gene fusions based on additional information provided by the Arriba output, such as the visual output of fusions, clinical data describing the samples (TCC, BCR, DoD, GGG), and the databases Mitelman DB [11], snoDB [34], and literature searches. The Mitelman DB is a database for chromosome aberrations and gene fusions in cancer, and it is updated quarterly. As of April 18, 2022, it contained 32,962 known gene fusions in cancer and 2,305 PCa-specific fusions. We chose the Mitelman DB as the primary gene fusion database because of its frequent update cycle. Other available gene fusion databases, such as FusionGDB2 [35], the TumorFusions database [36], ChimerDB4 [37], ChiTars5 [38], and Quiver [39], were not as up-to-date as the Mitelman DB. However, we searched all six available databases for the potential novel fusions as support. In addition to the gene fusion databases, we used the snoDB database to assess snoRNAs and their hosts that we found to be involved in gene fusions. The SnoDB is a database of human snoRNAs with data on their abundance, sequences, interactions, and host genes. It contained 1,970 snoRNA sequences in the database, version 1.1.0. To describe potential novel gene fusions, we relied on fusions detected in both discovery cohorts and matched all gene fusions identified in the discovery cohorts with the FFPE_Bx fusion set described above. Based on the assumption that fusions that occur in multiple cohorts are more likely to be true positives, we restricted the list to fusions that appeared in at least two cohorts. Finally, we ranked the matched fusions by the number of cohorts in which they were detected and if they could be found in the Mitelman DB. To include DKFZ_RP, we combined the above-described unfiltered set with all high-confidence gene fusions of DKFZ_RP and reduced the list to fusions detected in at least two different cohorts (Fig. S1).

### Statistical analyses

To relate gene fusions with PCa prognosis, we used Kaplan–Meier analyses, log-rank tests, and Cox regression in R 4.0.0, with the R packages survival (version 3.3–1, functions Surv(), coxph(), and survdiff()), rms (version 6.3–0, function npsurv()), and survminer (version 0.4.9) for visualization [40, 41]. We defined time to DoD as the primary endpoint for FF_RP and time to BCR as the prognostic endpoint for TCGA_PRAD, FFPE_Bx, and DKFZ_RP. It has been shown that BCR can be used as a surrogate endpoint for PCa; therefore, we can utilize both types of endpoints for our analyses [6, 42].

We conducted survival analyses for two different scenarios. First, we analyzed the impact of *TMPRSS2::ERG* fusion on the prognosis of PCa on a dichotomized scale (*TMPRSS2::ERG* fusion observed vs. not observed), as this fusion is ubiquitous and known to play a major role in PCa. We then analyzed the impact of a high number of gene fusions as a surrogate marker for genomic complexity on a dichotomized scale (less than the median of total fusions per cohort vs. more than or equal to the median of all fusions per cohort). Cox regression was used to investigate whether the number of detected gene fusions in a patient sample had an impact on prognosis after adjustment for the Gleason Grading Group (GGG). For this purpose, we performed Cox regression with fusion numbers dichotomized at the median and GGG (on a continuous scale).

## Results

This retrospective study was performed on transcriptome-wide sequencing data from four different primary PCa cohorts with long-term clinical follow-up data, information about the time to BCR or DoD as well as pathological GGG (Fig. 1). We used data of three general PCa cohorts, FF_RP, TCGA_PRAD, and FFPE_Bx, to identify gene fusions, including novel fusions, and annotated them using publicly available data. We also estimated the prognostic relevance of the detected gene fusions in primary PCa in comparison with clinical follow-up data. Using the DKFZ_RP cohort helped to evaluate our results from the perspective of early-onset PCa.

### Computational pipeline for gene fusion detection differentiates between tumor samples and samples without tumor tissue

**Fig. 2:**
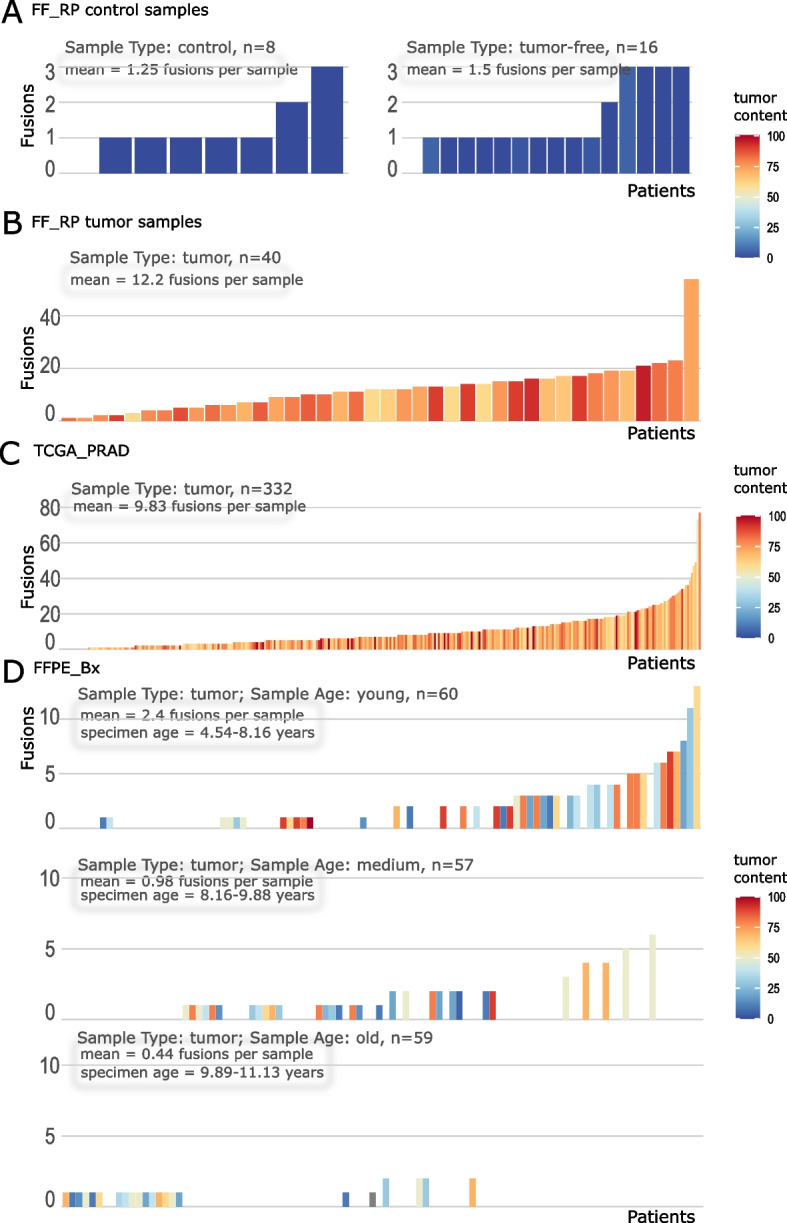
Numbers of high confidence gene fusions per sample. We plotted the numbers of gene fusions per sample, ordered by the number of fusions for each cohort. Samples are colored according to their TCC. For each plot, the mean number of fusions per sample is shown. **A** Bar plot of FF_RP control and tumor-free samples, respectively, and (**B**) FF_RP tumor samples. **C** Bar plot of the fusion number per sample for TCGA_PRAD. In (**D**), FFPE_Bx samples were split by specimen age (tertiles). The range of specimen ages is shown per age group

The FF_RP cohort allowed us to assess whether a difference in gene fusion numbers was evident between tumor and tumor-free samples. In total, we detected 633 gene fusions in 24 control samples (Table 1, column 1). According to Arriba's filtering criteria, 34 of these fusions had high confidence (5.37%, or 1.42 fusions per sample). The majority of gene fusions detected by Arriba were classified as medium (33.02%, 8.71 fusions per sample) or low confidence (65.56%, 17.29 fusions per sample), respectively (Table 1, column 1). The distribution of high-confidence fusions per control and tumor-free sample of FF_RP (Fig. 2A) revealed that both sample types had only low numbers of gene fusions per sample, with a mean of 1.25 (min = 0, max = 3) and 1.5 (min = 0, max = 3), respectively. In contrast, for the FF_RP tumor samples, we observed 488 high-confidence fusions (on average 12.20 fusions per sample, min = 1, max = 54; Fig. 2B). In total, 20.56% of the observed fusions were of high confidence (Table 1, column 2). Thus, consistent with our assumptions, we observed a higher rate of high-confidence fusions and a substantially higher number of fusions per tumor sample compared with specimens without tumor tissue.

**Table 1:** Numbers of detected gene fusions

		**FF_RP control**	**FF_RP tumor**	**TCGA_PRAD tumor**	**FFPE_Bx tumor**
	number of samples	24	40	332	176
all confidences	number of fusions	633	2,373	12,908	109,590
	average fusions per sample	26.38	59.33	38.88	622.67
	number of unique fusions per cohort	519	1,952	8,790	109,281
high	number of fusions (percentage of all confidences)	34 (5.37%)	488 (20.56%)	3,265 (25.29%)	230 (0.21%)
	mean fusions per sample	1.42	12.20	9.83	1.31
	number of unique fusions per cohort	31	447	3,072	204
medium	number of fusions (percentage of all confidences)	209 (33.02%)	752 (31.69%)	2,134 (16.53%)	7,476 (6.82%)
	mean fusions per sample	8.71	18.80	6.43	42.48
	number of unique fusions per cohort	159	567	1,778	7,440
low	number of fusions (percentage of all confidences)	415 (65.56%)	1,273 (53.65%)	8,002 (61.99%)	101,946 (93.02%)
	mean fusions per sample	17.29	31.83	24.10	579.24
	number of unique fusions per cohort	350	1,065	4,393	101,693

The columns depict the number of detected gene fusions per cohort. We report the numbers of control and tumor-free samples of FF_RP (column 1), the tumor samples of FF_RP (column 2), all samples of TCGA_PRAD (column 3), and all samples of FFPE_Bx (column 4). The rows depict the number of detected fusions, the corresponding mean of fusions per sample, and the unique occurrence of fusions across a cohort. The numbers are listed for the whole cohort (‘all confidences’) and separated for each Arriba confidence level (‘high’, ‘medium’, and ‘low’). Percentages in parentheses correspond to the proportion of each category compared to the total number of fusions per cohort. Percentages do not sum up to 100% because fusions can appear in multiple confidence groups

### The quality of detected gene fusions depends on the type of specimen conservation

In the 40 tumor samples from the FF_RP cohort, we detected 2,373 gene fusions (Table 1, column 2). Confidence filtering resulted in 20.56% high (mean = 12.2 fusions per sample), 31.69% medium, and 53.65% low confidence unique gene fusions (mean = 18.8 and 31.83 fusions per sample, respectively).

The fusion calling for the TCGA_PRAD cohort (*n* = 332) resulted in 12,908 gene fusions with 3,265 (25.29%, mean = 9.83 fusions per sample) high confidence, 2,134 (16.53%, mean = 6.43 fusions per sample) medium confidence, and 8,002 (61.99%, mean = 24.1 fusions per sample) low confidence fusions (Table 1, column 3). The distribution of fusions per sample for TCGA_PRAD revealed a minimum of 0 fusions and a maximum of 77 fusions (Fig. 2C).

In the 176 tumor samples from the third cohort, FFPE_Bx, we detected 109,590 gene fusions. Of these, only 230 (0.21%) were classified as “high confidence”, yielding 1.31 fusions per sample in the mean. 6.82% of the detected fusions of FFPE_Bx were labeled as “medium confidence” (mean = 42.48 fusions per sample), and the majority (93.02%) were considered low confidence (mean = 579.24 fusions per sample) (Table 1, column 4). With a mean value of 1.31 high confidence fusions per sample, the results for FFPE_Bx were lower than the number of fusions detected in the FF_RP control samples (mean = 1.42 fusions per sample). However, the values increased for FFPE_Bx with medium confidence fusions (mean = 42.48 fusions per sample versus mean = 8.71 fusions per sample in FF_RP controls, Table 1). To visualize the distribution of fusions per sample, we divided the cohort according to the age of the sample (Fig. 2D) by tertiles. We used this division to show the effect of storage time on the fusion detection in FFPE specimens. The storage time ranged from 4.5 to 11.1 years for the FFPE_Bx cohort. The results also revealed that FFPE_Bx samples conserved for less than 8.16 years carry more high confidence gene fusions (mean = 2.4 fusions per sample, min = 0, max = 13, Fig. 2D top plot) than older specimens (mean = 0.71 fusions per sample, min = 0, max = 6, Fig. 2D lower plots) (Wilcoxon rank sum test *p*-value = 5.6e^−06^, Fig. S2A).

Tumor samples from FF_RP and TCGA_PRAD resulted in similar proportions of confidence levels, whereas samples from FFPE_Bx showed a substantially lower number of high-confidence fusions and a larger number of low-confidence fusions (Table 1). In comparison, the means of fusions in the tumor samples of FF_RP and TCGA_PRAD were in the same range (12.2 and 9.83, respectively; Fig. 2B and C), whereas FFPE_Bx had a markedly lower mean of gene fusions per sample in all specimen age groups (means = 2.4, 0.94, and 0.44, respectively; Fig. 2D). This result was confirmed with a Wilcoxon rank-sum test. While the mean of TCGA_PRAD was only slightly lower than the mean of FF_RP (p-value = 0.01), the pairwise differences of means between FFPE_Bx and TCGA_PRAD or FF_RP yielded for both p-values of 2.2e^−16^ (Fig. S2B).

Interestingly, TCC was not related to the number of detected fusions per sample in all three cohorts (Fig. 2, coloring). Tumor samples from FF_RP and TCGA_PRAD all had a TCC of at least 50%, indicating that the effect of TCC on the number of fusions might not be detectable at such high TCC values (Fig. 2B and C). However, the cohort FFPE_Bx did not show an effect of TCC on the number of fusions, even though the TCC for biopsies ranged from 5 to 100%. This was evident for the youngest FFPE samples (Fig. 2D top), where some of the samples with the lowest TCC were ranked highest regarding the fusion numbers per sample. Overall, we observed a substantially lower number of high-confidence gene fusions in FFPE tissue specimens than in fresh frozen tissue. The difference was even more pronounced in old FFPE tissue specimens stored for more than eight years.

### Gene fusion detection pipeline shows good concordance with previously described ERG-fusions

We compared our findings for TCGA_PRAD with those reported by the TCGA consortium in 2015 [12] in order to assess the efficacy of the gene fusion detection method using Arriba. The consortium published the status of *ERG*, *ETV1*, *ETV4,* and *FLI1* fusions in the cBioportal database (dataset: Prostate Adenocarcinoma (TCGA, Cell 2015)) for 333 samples. Of these, 224 were part of the 332 TCGA_PRAD samples, fulfilling our inclusion criteria with respect to information on fusions, minimal TCC, and clinical follow-up (Fig. S3) as described by Kreuz et al. [6]. A direct comparison of the 224 samples revealed good concordance. In 35.7% (*n* = 80) of the samples, high-confidence *ERG*-fusions were detected with Arriba, while the TCGA consortium reported *ERG*-fusions in 27 additional samples (47.8% of the samples, Table S4A). However, the frequencies of medium-confidence fusions were consistent, with 46% (*n* = 103) of *ERG*-fusions detected in Arriba and 47.8% (*n* = 107) samples described by the TCGA consortium (Table S4A). Investigation of *ERG* expression in combination with *ERG* fusion status supported previous findings (Fig. S4). *ERG* expression commonly increases through fusion with androgen-dependent *TMPRSS2,* and we observed a bimodal distribution of expression in TCGA_PRAD (Fig. S4) [29]. For 23 of the 27 samples for which Arriba did not report high-confidence *ERG* fusion but high *ERG* expression (Fig. S4A), we detected a medium-confidence fusion of *ERG* with our pipeline (Fig. S4B). Of the four remaining samples for which we could not detect a fusion with Arriba, two samples exhibited high expression, while the other two exhibited low *ERG* expression (Fig. S4B). The calculation of contingency tables for *ETV1*, *ETV4*, and *FLI1* did not reveal such large variances between our results and those published by the TCGA consortium as for *ERG*. For *ETV1*, the consortium described 4.9% (*n* = 11) of fusion-affected samples, we detected 4.5% (*n* = 10) for high and high + medium confidence (Table S4B). For *ETV4,* we detected one more fusion (8 vs. 7 fusions) in the high + medium group than in the TCGA consortium, and we did not detect a *FLI1*-fusion, while the consortium detected two (Tables S4C and D).

Based on these findings, we draw the conclusion that the Arriba pipeline's results and those published by the TCGA consortium are consistent. A comparison of the frequencies of *ERG*, *ETV1*, *ETV4,* and *FLI1* fusions in TCGA_PRAD with those of FF_RP and FFPE_Bx (Table S5) supported our previous findings that far fewer gene fusions could be detected in FFPE_Bx. For instance, we detected 35.7% *ERG*-fusion-positive samples (high confidence) in TCGA_PRAD, 50% in FF_RP, and only 14.8% in FFPE_Bx (Table S5).

Nevertheless, we decided on a conservative approach to limit the number of false positives, using only high-confidence gene fusions for our analyses if not mentioned otherwise. Based on the lower quality of gene fusions observed for FFPE_Bx, we decided to use the cohorts FF_RP and TCGA_PRAD to identify novel fusion candidates and consult FFPE_Bx at all confidence levels to support our findings.

### The majority of detected fusions had not been previously reported

**Fig. 3:**
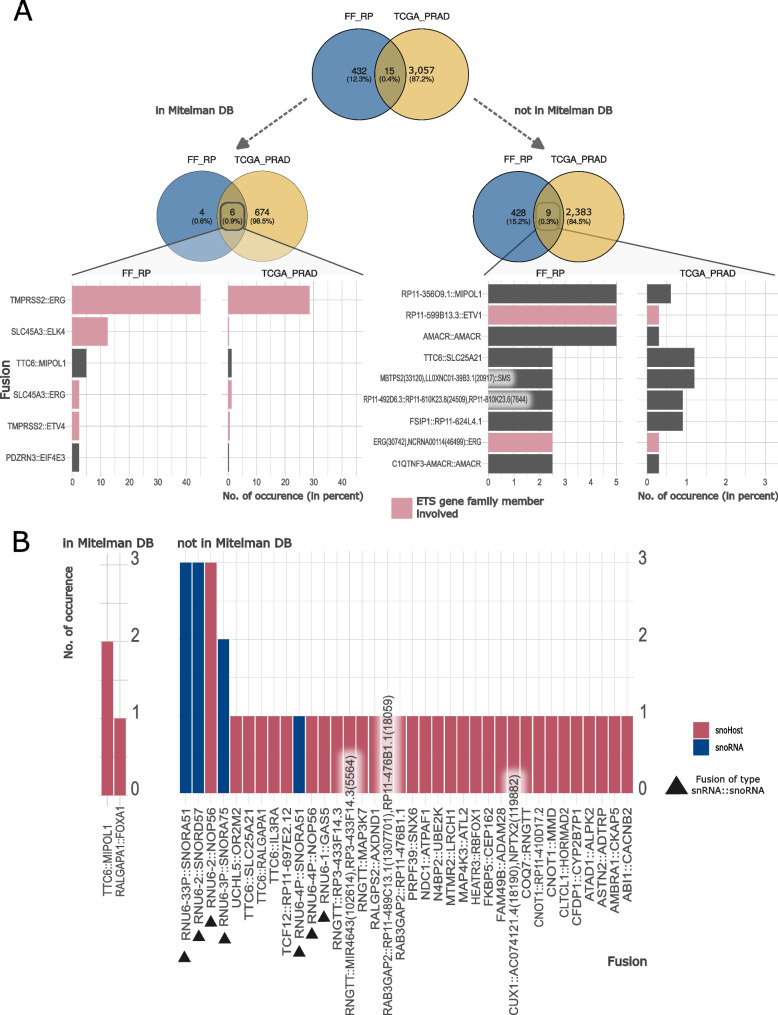
Characteristics of detected high confidence fusions for discovery cohorts FF_RP and TCGA_PRAD. **A** The Venn diagrams show the overlap of gene fusions between the two cohorts for all fusions (top), those fusions that are described in the Mitelman DB (left), as well as those that are not described (right). The overlap of fusions are shown in bar plots below, with their frequency in percent. Red: gene fusions that involve genes from the ETS family. Plot (**B**) shows the occurrence of gene fusions in FF_RP that involve snoRNAs (blue) or their host genes (red). Triangles highlight fusions of the type snRNA::snoRNA/host gene

The two cohorts, TCGA_PRAD and FF_RP, served as discovery cohorts for the identification of gene fusions, as both consisted of fresh-frozen samples. In summary, we identified 3,504 unique high-confidence fusions in both cohorts (Fig. 3A, Table S6). Only a small number of 15 fusions appeared in both cohorts (0.4% of all unique high-confidence fusions, Fig. 3A, Table S7). Matching all fusions with annotated gene fusions in the Mitelman DB, we observed a large fraction (*n* = 2,820) of still unknown gene fusions, labeled with “high confidence” by Arriba (Fig. 3A). The detected fusions described in the Mitelman DB (*n* = 684) included the most common gene fusions, such as *TMPRSS2::ERG* and *SLC45A3::ELK4*. *TMPRSS2::ERG* was the most frequent fusion in both cohorts, detected in 45% of FF_RP tumor samples and 28.6% of TCGA_PRAD samples. These numbers differ from the *ERG*-fusion frequencies described above because of the reduction to only *TMPRSS2::ERG* fusions and the inclusion of 108 samples, which matched our inclusion criteria but had no matching results published by the TCGA consortium.

The second most frequently known gene fusion was *SLC45A3::ELK4* in more than 10% of FF_RP samples and approximately 1% of TCGA_PRAD samples (Fig. 3A, left). Both fusion partners are known to be involved in gene fusions in PCa; *SLC45A3* is specifically expressed in the prostate, while *ELK4* is a member of the ETS gene family, similar to *ERG *[16, 43, 44]. In addition to *ELK4* and *ERG*, multiple *ETS*-family members were detected in gene fusions (Fig. 3A, red bars). Another known gene fusion detected in multiple samples is *TTC6::MIPOL1*. *MIPOL1* is thought to act as a tumor suppressor, and its fusions may accompany *ETV1* fusions [45]. *TTC6* is a snoRNA host gene located on chromosome 14 and is specifically expressed in breast and prostate tissues [34, 46]. The *TTC6::MIPOL1* fusion has been described for both tissue types [47, 48].

Among the group of most frequent, not yet described fusions were also multiple *ETS*-family genes (Fig. 3A, right side, red bars). Another group of genes frequently represented in the detected fusions of both cohorts were genes with an "RP11" prefix (Fig. 3A, right side) [49]. These are genes derived from the BAC clone library at the Roswell Park Cancer Institute, without approved gene symbols. "RP11" is the identifier of the clone [50]. One of these genes, *RP11-356O9.1,* is annotated as a lncRNA on chr14 that is predominantly expressed in the prostate, and a fusion with *ETV1* has been described previously in the PCa cell line MDA-PCa 2B [51, 52]. In our case, *RP11-356O9.1* frequently fused with *MIPOL1,* as well as less frequently with *ETV1*, *TTC6*, and *YME1L1* (Table S6). *MIPOL1* and *RP11-356O9.1* are adjacent genes on chromosome 14. This suggests that these cases are read-through transcription gene fusions. In addition, *ETV1* has been described as being translocated to chr14 in that region in PCa, and through this translocation, another read-through fusion can be formed [51].

In FF_RP, we also detected a group of fusions between snRNAs (prefix "RNU" [53]) and snoRNAs (prefix "SNOR") in multiple samples. Upon further inspection and reconciliation with the snoDB [34], we revealed various fusions involving snoRNAs and their host genes in FF_RP (Fig. 3B, Fig. S5). Only two of the fusions have been previously described (Fig. 3B, left panel), while the remaining 36 fusions were not included in the Mitelman DB. Interestingly, snRNAs were involved in 7 of the 36 snoRNA/host gene fusions (Fig. 3B, triangles). We found 209 gene fusions in TCGA_PRAD that involved a snoRNA host. Two of these fusions, *TTC6::MIPOL1* and *TTC6::SLC25A21*, were found in both discovery cohorts (Tables S6 and S7). Fusions with snoRNAs could not be detected in TCGA_PRAD, as snoRNAs carry no poly(A) tails and are therefore not processed in poly(A)-RNA sequencing. Background information on these types of fusions is scarce; however, Persson et al. [49] recently described gene fusions in breast cancer involving snoRNAs, disrupting snoRNA/host gene transcriptional balance, and contributing to a change in the expression of snoRNAs. Such fusions have not yet been described for PCa.

Revisiting the FF_RP tumor-free and BPH samples revealed gene fusions of the type snRNA::snoRNA in all sample types; however, the control samples had fewer supporting reads and a lower confidence level than the tumor samples (Table S8).

To evaluate whether these fusions were true positives or artifacts, we performed polymerase chain reaction (PCR) analyses. For *TMPRSS2::ERG*, our positive control, PCR amplified a product with the expected product size (313 bp) in the sample where we predicted a fusion using Arriba (Fig. S6A, lane 5). Interestingly, the three different snRNA::snoRNA fusions that we wanted to verify showed bands with the expected sizes (103 bp, 106 bp, and 113 bp, respectively), but also for those samples we did not predict a fusion for (Fig. S6B-D).

### Tumor suppressor genes are highly represented in gene fusions

Since the literature describes that most genes are unlikely to be partners in just one specific fusion but rather are promiscuous [8], we also inspected the genes that are frequently involved in different gene fusions in our cohorts.

**Fig. 4:**
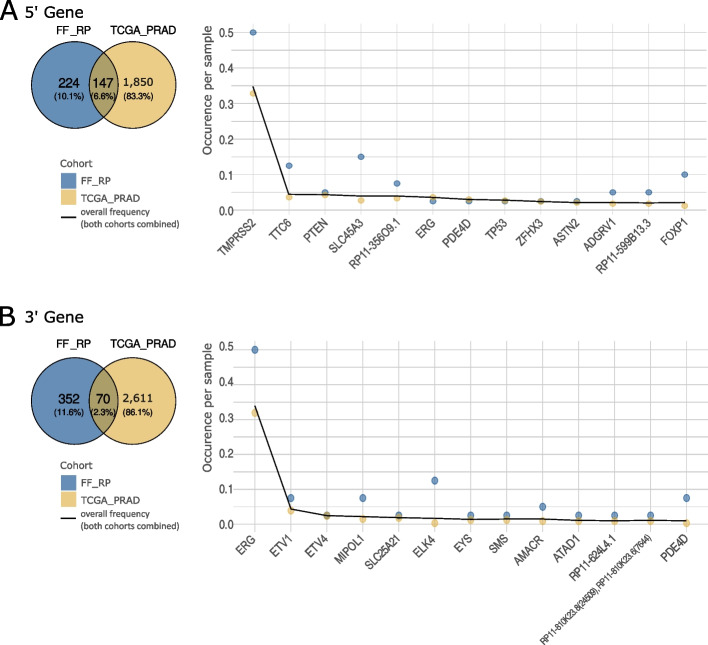
Genes most frequently involved in gene fusions in the cohorts FF_RP and TCGA_PRAD. **A** Results for the 5’ gene and (**B**) results for the 3’ gene. The Venn diagrams show the numbers of different genes involved in gene fusions as well as their overlap between the two cohorts. Plots show the number of occurrences of genes found in both cohorts, divided by sample size for FF_RP (blue, *n* = 40) and TCGA_PRAD (yellow, *n* = 332). The black lines depict combined values calculated as the number of occurrences in FF_RP plus the number of occurrences in TCGA_PRAD, divided by the sum of the sample sizes of both cohorts. This value was used to sort the genes

The most commonly affected genes among known fusions are *TMPRSS2* as 5’ gene (Fig. 4A) and *ERG* as 3’ gene (Fig. 4B), based on their ubiquitous fusion in PCa. Among the genes that are frequent but not involved in one specific fusion, prominent tumor suppressors such as *FOXP1*, *PTEN,* and *TP53* as 5’ genes (Fig. 4A) stand out. The gene *PTEN* was involved in several fusions in FFPE_Bx and TCGA_PRAD. In addition to its tumor suppressing functionality, it is known to be involved in apoptosis and neurogenesis. Its general expression is cancer unspecific, however in PCa a decrease in expression is linked to the *TMPRSS2::ERG* fusion [54–56]. *PTEN* gene fusions have also been found in several other primary tumors [9]. *FOXP1* expression has a low tissue specificity, the protein acts as transcriptional repressor and can negatively regulate AR signaling [55, 56]. The product of *TP53* is ubiquitously expressed and involved in multiple tumor suppressing pathways such as growth arrest or apoptosis [55, 56]. Combining ERG overexpression with *PTEN* or *TP53* loss — that is, through fusion or deletion — can induce cell migration as well as promote the development and progression of PCa [18, 20, 57].

The most frequent 3’ genes were also among the most frequent fusions (Fig. 4B). These were genes of the RP11 group, with *RP11-356O9.1* represented in both cohorts, as well as ETS-family genes such as *ELK4, ETV1,* or *ETV4 (*Fig. 4B*).* Gene set enrichment analyses of genes involved in fusions in the discovery cohorts confirmed our findings of a higher involvement of tumor suppressor genes in fusions (*p* = 0.013, adjusted with the Benjamini–Hochberg procedure, Fig. S7) and a significant enrichment of genes from the MsigDB's androgen response hallmark set (*p* = 0.008, adjusted with the Benjamini–Hochberg procedure, Fig. S7) [58].

### Detected gene fusions in discovery cohorts are partly detectable in FFPE biopsies

Of the 3,504 high-confidence fusions detected in fresh-frozen tissue of the two discovery cohorts (shown in Fig. 3A), we identified 36 gene fusions that occurred in at least two cohorts (FF_RP or TCGA_PRAD (high confidence) or FFPE_Bx (all confidences)). Nine gene fusions were found in all three cohorts (Table 2). Of these, five gene fusions have already been described in the literature and can therefore be found in the Mitelman DB. Of the four gene fusions that were not listed in the Mitelman DB, *ERG::ERG*, and *FSIP1::RP11-624L4.1* have been described elsewhere [59, 60] (Table 2, top four rows). Next, we searched the other databases, FusionGDB2, TumorFusions database, ChimerDB4, ChiTars5, and Quiver, for the two remaining potential novel gene fusions, *AMACR::AMACR* and *MBTPS2*, *LL0XNC01-39B3.1*::*SMS,* without results. Although *AMACR* is known to be involved in oncogenic gene fusions in PCa, a fusion with itself has not yet been described [61]. The three genes involved in the fourth fusion, *MBTPS2*, *LL0XNC01-39B3.1*, and *SMS,* are not known to be related to any diseases in the prostate or gene fusions, but since all three genes are neighboring genes (Fig. S8), read-through transcription was likely. In addition, we observed higher expression of SMS in luminal cells in the PCa Cell Atlas [21] than in the other cell compartments (Fig. S9A-C), as well as a significant correlation between high *SMS* expression and fusion involvement (Wilcoxon, *p* = 0.028, Fig. S9D).

**Table 2:** Overlap of fusions between the three cohorts as well as their occurrence in Mitelman DB

Fusion	FFPE_Bx	FF_RP	TCGA_PRAD	Known
ERG(30,742),NCRNA00114(46,499)::ERG	1	1	1	FALSE
AMACR::AMACR	1	1	1	FALSE
FSIP1::RP11-624L4.1	1	1	1	FALSE
MBTPS2(33,120),LL0XNC01-39B3.1(20,917)::SMS	1	1	1	FALSE
AMBRA1::CKAP5	1	1	0	FALSE
THBS1::RP11-624L4.1	1	1	0	FALSE
LINC00506(49,329),MIR4795(19,791)::CHMP2B	1	1	0	FALSE
RP11-159H20.3(25,945),FOXB2(602)::PRUNE2	1	1	0	FALSE
RP11-597A11.11::RP11-597A11.1	1	1	0	FALSE
AC004921.2(27,972),PTPN12(47,164)::GSAP	1	1	0	FALSE
RP11-356O9.1::ETV1	1	0	1	FALSE
PTEN::RP11-380G5.4(8171),RP11-129G17.2(201,135)	1	0	1	FALSE
PTEN::RNLS	1	0	1	FALSE
MAPKAPK5::ACAD10	1	0	1	FALSE
ACPP::CPNE4	1	0	1	FALSE
CTC-340A15.2::CTC-535M15.2	1	0	1	FALSE
PLPP1::SKIV2L2	1	0	1	FALSE
SCHLAP1::UBE2E3	1	0	1	FALSE
RP11-17A19.1::KCTD1	1	0	1	FALSE
ZFHX3::AC004158.2	1	0	1	FALSE
RP11-356O9.1::MIPOL1	0	1	1	FALSE
TTC6::SLC25A21	0	1	1	FALSE
RP11-599B13.3::ETV1	0	1	1	FALSE
C1QTNF3-AMACR::AMACR	0	1	1	FALSE
RP11-492D6.3::RP11-810K23.8(24,509),RP11-810K23.6(7644)	0	1	1	FALSE
SLC45A3::ELK4	1	1	1	TRUE
TMPRSS2::ERG	1	1	1	TRUE
TMPRSS2::ETV4	1	1	1	TRUE
TTC6::MIPOL1	1	1	1	TRUE
SLC45A3::ERG	1	1	1	TRUE
ERG::TMPRSS2	1	0	1	TRUE
PMEPA1::ETV4	1	0	1	TRUE
SLC45A3::ETV1	1	0	1	TRUE
IQSEC1::SCCPDH	1	0	1	TRUE
GPATCH8::PYY	1	0	1	TRUE
PDZRN3::EIF4E3	0	1	1	TRUE

The rows show 36 gene fusions found in at least two cohorts. Columns 2–4 show the detection status of the fusion per cohort, where 1 means fusion can be detected and 0 means fusion cannot be detected. Column 5 states whether the fusion can be found in the Mitelman DB (TRUE) or not (FALSE). The table is gradually sorted by prominence according to the Mitelman DB (column 5) and the sum of cohorts in which a fusion can be found, as well as their rediscovery in FFPE_Bx

**Fig. 5:**
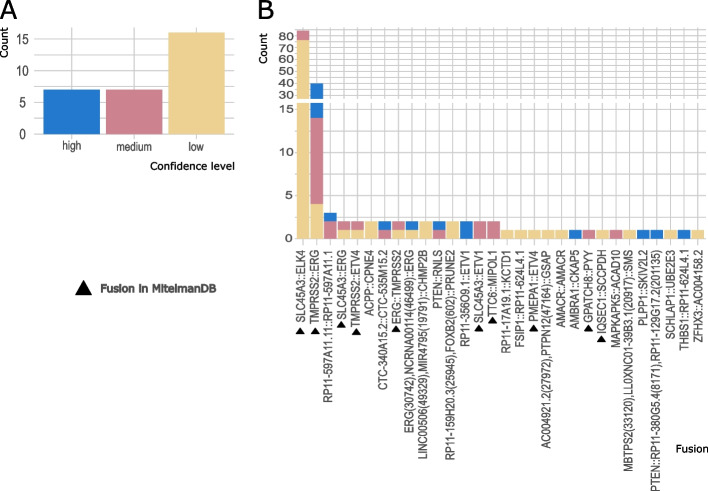
Confirmation of gene fusions in FFPE_Bx. Unique gene fusions of discovery cohorts FF_RP and TCGA_PRAD that have been detected in FFPE_Bx. **A** Distribution of the 30 rediscovered gene fusions per Arriba confidence levels. **B** Histogram of the numbers of samples in which each gene fusion could be detected. Bars are colored according to confidence level. If a fusion was detected multiple times in one sample, the highest confidence level was assumed, and only one occurrence per sample was counted. Triangles next to the fusion names indicate whether a fusion can be found in the Mitelman DB

Figure 5 provides more detailed information on gene fusions from the discovery cohorts that could be detected in FFPE_Bx (Table S9). Of the 36 fusions described above, we detected a total of 30 fusions in FFPE_Bx (Fig. 5, Table 2). Of these 30 gene fusions, 16 were classified as low confidence and seven as high and medium confidence, respectively (Fig. 5A). The most prominent confirmed gene fusions were *TMPRSS2::ERG* and *SLC45A3::ELK4* (Fig. 5B). Both are well-known gene fusions associated with PCa; however, the high quantity of low-confidence *SLC45A3::ELK4* fusions in FFPE_Bx suggests that false positive hits were detected. All other gene fusions were detected in a small number of samples. Among these fusions were *TMPRSS2* and *SLC45A3* fusions with other members of the ETS gene family, such as *ETV1* and *ETV4,* which are also known to be involved in PCa gene fusions (Fig. 5B). The 30 fusions in FFPE_Bx corresponded to 10 known and 20 unknown fusions when comparing the results with the Mitelman DB (Fig. 5B, triangles).

### Number of gene fusions partly serves as an additional prognostic factor for PCa

After quality control of the cohorts and characterization of differences in fusion calling quality between them, we also analyzed the impact of gene fusions on the prognosis of PCa patients.

**Fig. 6:**
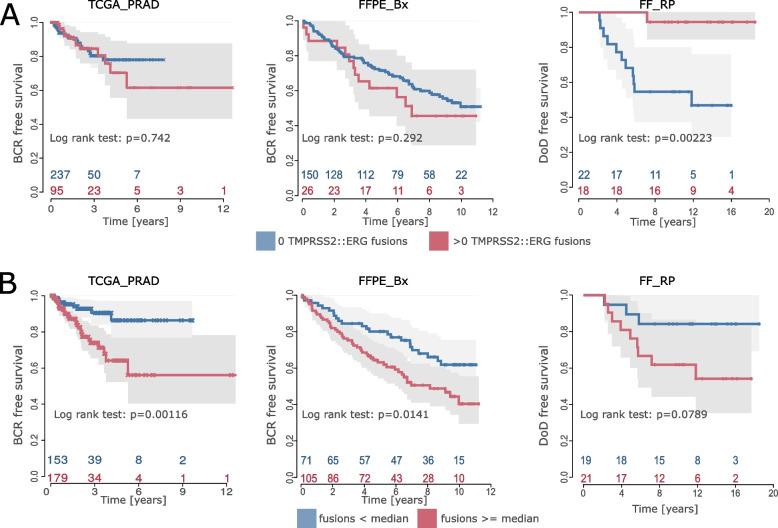
Prognosis of PCa progression. **A** Prognosis of PCa for all three cohorts for patients with and without TMPRSS2::ERG fusion. Kaplan–Meier curves and log-rank tests for TCGA_PRAD with *n* = 332 and 42 events (BCR), as well as FFPE_Bx with *n* = 176 and 75 events (BCR), and FF_RP with *n* = 40 and 12 events (DoD). **B** Kaplan–Meier curves and log-rank tests for TCGA_PRAD (high confidence gene fusions), FFPE_Bx (combined fusions), and FF_RP (high confidence, left to right), grouped by the median of the total number of gene fusions (< median of fusions per sample vs. ≥ median of fusions per sample)

To see if the most common PCa fusion, *TMPRSS2::ERG*, had a direct effect on PCa prognosis, we used Kaplan–Meier curves and log-rank tests with the samples grouped by *TMPRSS2::ERG* status (*TMPRSS2::ERG* fusion observed vs. not observed). Analyses with FF_RP (*n* = 40, 12 events) resulted in a significantly better prognosis for patients with the *TMPRSS2::ERG* fusion (Fig. 6A, right panel, *p* = 0.00223). The larger cohorts, TCGA_PRAD (*n* = 332, 42 events) and FFPE_Bx (*n* = 176, 75 events), showed an effect towards a worse prognosis for patients who carried a *TMPRSS2::ERG* fusion. However, the log-rank tests for both cohorts were not significant (Fig. 6A, left panel, *p* = 0.742, middle panel, *p* = 0.292). These inconsistent results suggest that *TMPRSS2::ERG*, although ubiquitous in PCa, is not a suitable prognostic marker.

Next, we considered all detected gene fusions (high-confidence fusions for TCGA_PRAD and FF_RP, and the combined set of fusions for FFPE_Bx (Fig. S1)), dichotomizing the cohorts according to the median total number of fusions per sample. All three cohorts had an adverse prognosis in the group with more fusions. The log-rank test for TCGA_PRAD with 332 samples and 42 BCR events showed a significant difference (*p* < 0.01, median = 7 fusions, 5-year BCR-free survival 86.3% vs. 64.1%) in the time to BCR between patients (Fig. 6B, left). In addition, the calculation of Kaplan–Meier estimates of the confirmation cohort FFPE_Bx (Fig. 6B, middle) also resulted in a significantly shorter time to BCR (*p* = 0.0141, median = 2 fusions, 5-year BCR-free survival 80.1% vs. 63.1%) for patients with a higher number of gene fusions. Only FF_RP with 40 tumor samples and 12 DoD events presented a non-significant association (*p* = 0.0789*,* median = 12 fusions, 5-year survival 90% vs. 75%), but still supported the trend that the other two cohorts exhibited (Fig. 6B right).

As a next step, we assessed whether gene fusions remained an independent prognostic factor when considering histological classification by GGG. To address this, we performed multivariate Cox regression with the total number of fusions per patient (dichotomized at the median) and the associated GGG (Table 3). The regression analysis showed that, in line with the Kaplan–Meier curves above, the number of fusions was significantly associated with prognosis in addition to GGG for TCGA_PRAD (*p* < 0.05, Table 3) and FFPE_Bx (*p* < 0.05, Table 3). However, in FF_RP, the number of fusions was not significantly associated with the prognosis (*p* = 0.46, Table 3). Multivariate Cox regression with the additional inclusion of an established prognostic transcriptome score for localized PCa, the revised ProstaTrend Score [21], provides only weak evidence that a model combining GGG and a prognostic transcriptomic score with the overall number of observed gene fusions improves predictive performance (Table S10). For FFPE_Bx, gene fusions and GGG were significant (p_Genefusions_ < 0.05, p_GleasonGrading_ < 0.001), whereas the revised ProstaTrend score was not (p_ProstaTrend_ = 0.3, Table S10). In TCGA_PRAD, GGG was significant (*p* < 0.01), while the number of fusions and revised ProstaTrend Score both had a p-value slightly above 0.05 (both *p* = 0.058, Table S10). FF_RP showed non-significant values for fusion numbers and GGG (*p* = 0.16 and *p* = 0.19, respectively), but a significant influence of the revised ProstaTrend score on survival (*p* = 0.002). For all three cohorts, we observed that the histological GGG and transcription-based revised ProstaTrend score were correlated, while the number of fusions seemed to be an independent marker in comparison with GGG (Fig. S10).

**Table 3:** Multivariate Cox regression for all three cohorts

		**TCGA_PRAD (*n* = 332, e = 42)**	**FFPE_Bx (combined) (*n* = 176, e = 75)**	**FF_RP (*n* = 40, e = 12)**
total fusions	median	7	2	12
	logHR (95% CI)	0.82 (0.13, 1.5)	0.53 (0.07, 0.99)	-0.46 (-1.66, 0.74)
	*p*	0.0198	0.0236	0.45564
GGG	logHR (95% CI)	0.62 (0.32, 0.92)	0.67 (0.49, 0.85)	0.79 (0.28, 1.3)
	*p*	4.79e-05	1.72e-13	0.00238

Multivariate Cox regression with dichotomized numbers of fusions and continuous GGG. Columns represent the results per cohort, with all included TCGA_PRAD samples (*n* = 332, column 1), the combined FFPE_Bx dataset (*n* = 176, column 2), and the FF_RP tumor samples (*n* = 40, column 3). The rows represent the tested variables with their logHR, 95% confidence interval, and *p*-value. For the variable of total fusions, the median per dataset is recorded in the uppermost row

*logHR* Logarithmic hazard ratio, *CI* Confidence interval, *p p*value

### Early-onset PCa cohort supports potential novel gene fusions

We used samples from 82 patients with early-onset PCa to evaluate our previous findings. We wanted to determine, if the potential novel gene fusions we discovered, as well as the findings of a trend toward a worse prognosis with a higher number of gene fusions, could be confirmed in early-onset PCa despite known molecular differences [20].

With our general gene fusion pipeline used above, Arriba reported 2,168 gene fusions of all confidence levels for DKFZ_RP, with an average of 26.44 fusions per sample (Table S13A). The distribution of fusions per confidence level was similar to the distributions in the cohorts TCGA_PRAD and FF_RP, with 33.03% high confidence fusions, 19.37% medium confidence fusions, and 53.41% low confidence fusions (Table 1, Fig. S11A, and Table S13A, respectively). As expected, the most frequent fusion was *TMPRSS2::ERG*, which was detected with high confidence in 54.88% of samples (Table S11). In addition, *ERG* was a more frequent fusion partner in the DKFZ_RP cohort (58.5%, Table S5) than in the other cohorts (35.7–50%, Table S5), whereas fusions with *ETV1*, *ETV4*, and *FLI1* did not show an increased incidence (4.9% vs. 2.3–7.5% *ETV1*, 2.4% vs. 0–3.1% *ETV4,* and 0% vs. 0% *FLI1*, Table S5). Overlapping the high-confidence fusions detected in the DKFZ_RP cohort with those in the other cohorts resulted in 52 fusions that could be detected in at least two cohorts. Of the four above-described fusions that have not been previously described in the Mitelman DB, we detected three fusions in the DKFZ_RP cohort: *ERG::ERG*, *AMACR::AMACR*, and *MBTTPS2,L0XNC01::SMS* (Table S12).

Finally, we performed survival analyses with high-confidence fusions detected in the DKFZ_RP dataset. Kaplan–Meier curves and log-rank tests based on the number of TMPRSS2::ERG positive samples (TMPRSS2::ERG fusion observed vs. not observed) showed that patients with a fusion had a significantly better prognosis (*p* = 0.00643, 5-year BCR-free survival 86.1% with fusion vs. 64.5% without, Fig. S11B). In contrast, analyses with the total number of fusions per patient resulted in a non-significant worsening of prognosis for patients with above median fusions counts (median = 6 fusions, *p* = 0.0755, 5-year BCR-free survival 62.8% with fusion vs. 87.2% without, Fig. S11C). Cox regression analysis with dichotomized total fusion number (*p* = 0.482) and continuous GGG (*p* = 0.000104) supported these findings (Table S13B).

## Discussion

With the detection and characterization of gene fusions from total RNA-sequencing and poly(A)-RNA-sequencing of four datasets (*n* = 630 samples), we identified two potential novel gene fusions and gained insights into PCa prognosis.

The first aim of our study was to characterize the gene fusions we identified in the available cohorts. The majority of fusions we encountered were not reported in the Mitelman DB, meaning that they are not yet (sufficiently) described. This may be due to the low prevalence of specific fusions. Another reason could be that fusions described in the literature were mainly detected in studies using poly(A)-enriched sequencing (i.e., mRNA-Seq). In contrast, our study also included two cohorts sequenced using rRNA-depleted total RNA-Seq, of which one was sequenced with approximately 200 million reads per sample.

However, as expected, *TMPRSS2::ERG*, which occurs in approximately 50% of PCa cases [62], was the most frequent fusion detected with high confidence in all cohorts. Besides *ERG*, we observed plenty of fusions with other *ETS*-family members as 3' partners, such as *ETV1*, *ETV4*, or *ELK4*. The *ETS*-family of genes consists in humans of 29 transcription factors that are involved in a variety of cellular functions along with gene fusions in various cancers [63]. Of the fusions known in PCa with partners such as *TMPRSS2* or *SLC45A3*, a gene that codes for a transporter protein and is enriched in PCa [55, 56], we could also identify fusions affecting *ETS*-family members that have not previously been described in PCa (Table 2).

Overall, we found that tumor suppressor genes and genes related to the MsigDB androgen response hallmark set were significantly enriched in fusions. This was in line with the role of gene fusions in cancer development [64] as well as the important role of androgen regulation in PCa in particular [22].

Among the most frequent fusions detected in the deeply sequenced total RNA FF_RP cohort, another group of fusions was noticeable: fusions of snRNA genes with snoRNA genes. On closer inspection, we found multiple fusions with snoRNA host genes, such as *TTC6*, in all cohorts. Arriba found fusions with snoRNAs in FF_RP and FFPE_Bx. In TCGA_PRAD, fusions with snoRNAs were not detectable, as the sequencing protocol only covered RNA molecules with a poly(A)-tail [12]. However, we also identified fusions between snRNAs and snoRNAs in control samples of FF_RP. Thus, it is currently unclear whether the observed fusions with snRNAs or snoRNAs are artifacts or true gene fusions associated with prostate tissue but not PCa.

Despite the low prevalence of most gene fusions, we also identified three candidates that could be detected in all four cohorts but were not described in the Mitelman DB. Of these three gene fusions, *ERG::ERG* could be confirmed as known fusion with literature research [59], whereas *AMACR::AMACR* and *MBTPS2,LL0XNC01-39B3.1::SMS* have not yet been described in the literature. *AMACR*, an isomerase with enriched expression in liver, renal, and prostate cancer [55, 56], has been described in PCa fusions but not with itself; however, the fusion of *ERG* with itself is known to occur in PCa. Therefore, this could likely be a similar event acting as a feedback loop with itself [11]. *MBTPS2* and *SMS* are neighboring genes. The gene *MBTPS2* codes for a protease that is related to the steroid metabolism and ER stress response. It shows a low cancer specificity and its prognostic value is unknown [55, 56]. The protein of *SMS* catalyzes the production of spermine and is by default expressed in the prostate [55, 56]. The non-coding RNA *LL0XNC01-39B3.1* can be found between the two genes Arriba named both *MBTPS2* and *LL0XNC01-39B3.1* as the fusion partners of *SMS,* and the read coverage of samples carrying the fusion was slightly higher in the non-coding area (Fig. S8). None of the three genes are known to have a specific role in PCa development; however, *SMS* is part of the androgen response hallmark set of MsigDB [58]. Using single-cell sequencing data, we observed high expression of *SMS* in luminal cells, which is the cell type where PCa is thought to originate alongside basal cells [22] (Fig. S9). According to StringDB [65], the proteins of genes do not normally interact. Thus, it is unclear which function this fusion could provide for PCa and whether it is a tumorigenic event, a bystander aberration, or a false-positive finding. On the other hand, we observed increased gene expression of *SMS* in fusion-positive samples, and the fusion was one of the very few gene fusions that we could detect in all cohorts (Fig. S9D). Therefore, the *MBTPS2/LL0XNC01-39B3.1::SMS* fusion was likely a true positive.

In all our characterizations, most gene fusions occurred very infrequently, which makes the investigation of biological and clinical relevance statistically challenging. A proposal by Persson et al. [49] to cluster gene fusions by the functionality of the involved genes rather than considering individual genes seems to be a promising method to mitigate the restrictions of cohort size and the low frequency of single fusions. This hypothesis should be tested in the future, considering the similarity of the gene expression landscapes of samples harboring these fusions.

The second aim of our study was to investigate the prognostic relevance of gene fusions. Performing survival analyses with samples dichotomized by *TMPRSS2::ERG* occurrence underlined the varying descriptions of the prognostic role of this fusion in the literature [66]. This is in line with the findings of Song and Chen in 2018 [67] that *TMPRSS2::ERG* is not associated with BCR or DoD, which are the endpoints available for our study. Also in line with their findings is our observation of a higher number of *TMPRSS2::ERG* fusions in the young patients of the DKFZ_RP cohort in comparison with the other three cohorts. In one of their meta-analyses, Song and Chen examined eight published studies regarding the relationship between the age of PCa patients and *TMPRSS2::ERG* fusions. They reported a significant increase in fusions in younger patients (age ≤ 65 years). Overall, our prognostic analyses exhibited considerable heterogeneity, from a highly significant better prognosis for *TMPRSS2::ERG*-fusion positive patients in the cohorts FF_RP and DKFZ_RP to no specific effect in the cohorts TCGA_PRAD and FFPE_Bx.

In analyses based on the overall number of detected fusions, we observed a significantly adverse prognosis in two out of four cohorts (TCGA_PRAD and FFPE_Bx), as well as a consistent trend for FF_RP and DKFZ_RP for patients with a high number of gene fusions. However, the small cohort size (*n* = 40 tumor samples) and accompanying low number of events (*n* = 12 events) of FF_RP limited its statistical power. DKFZ_RP was a special cohort because it described early-onset PCa, whereas the other three cohorts did not focus on a specific PCa subtype. We observed that for two out of four cohorts, the number of gene fusions had an additional prognostic value beyond that of GGG (multivariate Cox regression). Gerhauser et al. [20] described molecular differences between early- and late-onset PCa. Accordingly, the influence of familiar predisposition in early-onset PCa [22] and the relatively low number of events (*n* = 18) could influence the prognosis and explain the differing results between the DKFZ_RP cohort and the TCGA_PRAD and FFPE_Bx cohorts. We hypothesize that gene fusions are a measure of genomic instability or cellular disorganization induced by PCa [10]. Genomic instability and heterogeneity have been described as being associated with a worse prognosis of PCa [22]. However, measuring gene fusions transcriptome-wide is costly. Currentlyavailable gene fusion panels use targeted approaches and cannot account for the total number of gene fusions in a sample [68]. In addition, it is difficult to transfer the threshold of fusion numbers between cohorts, which presents challenges for the translation of the marker to usability in clinical practice. Furthermore, the prognostic differences between the considered groups were of moderate effect size and varied considerably between the cohorts.

Our analyses revealed some limitations in the detection of gene fusions related to the composition and conservation of the cohorts. First, the quality of RNA has a major impact on the quality of detected gene fusions. As is known for FFPE samples, the quality of the RNA decreases with the age of the specimen [69]. The different distribution of detected gene fusions by confidence level in the FFPE_Bx cohort compared to the fresh-frozen tissue specimen cohorts (FF_RP, TCGA_PRAD, and DKFZ_RP) suggests that the level of RNA degradation in FFPE tissue specimens affects how easily gene fusions can be found. We observed a shift towards low confidence fusions in FFPE tissue: likely true positive fusions lacked supporting reads, and more false positive fusions were called due to degradation. Low RNA quality and degradation of RNA complicated identification and likely resulted in false-positive fusions. We showed that this decline in quality affected the detection of gene fusions, with a clear association between specimen age and the number of detected fusions as well as major differences between fresh-frozen and FFPE samples.

The second limiation that became apparent in this study was the number of samples in a cohort. The sample size of the TCGA_PRAD cohort (*n* = 332) allowed the description of a broad spectrum of gene fusions in PCa and their frequencies, while the FF_RP cohort revealed only a limited spectrum of gene fusions present in PCa. In contrast, the mean number of fusions per sample was comparable between cohorts with a larger sample size, such as TCGA_PRAD, and cohorts with a smaller sample size, such as FF_RP (*n* = 40). The fusion numbers of FFPE_Bx were of no weight for this finding due to the poor RNA quality of the cohort. Another limiting factor for analyses with FF_RP was the low number of events (e = 12 DoD), resulting in low statistical power to assess the correlation between gene fusions and prognosis, thus restricting the survival analyses with this cohort.

## Conclusions

In this study, we identified two novel gene fusions for PCa that have not yet been described in the literature but were detectable in all cohorts included in our study. In addition, we provided information on the prognostic relevance of gene fusions in primary PCa. We found evidence that the overall number of gene fusions in PCa tissue specimens was related to the prognosis of the disease in two cohorts, even when adjusting for GGG. However, the prognostic effect varied between the cohorts. The small number of events in two of the four cohorts as well as the specimen age of the FFPE samples limited our analysis. For FFPE-preserved samples, it is advisable to use fresh specimens for fusion detection to avoid loss of quality. Thus, we conclude that the overall number of gene fusions as surrogate marker for the degree of genomic instability is not a suitable parameter for inclusion in statistical models for PCa prognosis at this time point.

## Supplementary Information

**Additional file 1**: This document contains additional tables (Tables S1–S13) and figures (Figures S1–S11) to support the findings of our study. Tables and figures that are too extensive are provided as separate files; their captions, however, can be found here. Extra files provided: **Table S3.** Whitelist of known fusions as used with Arriba. **Table S6.** High confidence gene fusions of TCGA_PRAD and FF_RP. **Table S7.** Overlap between discovery cohorts TCGA_PRAD and FF_RP. **Table S8.** snoRNA gene fusions detected in all samples of FF_RP. **Table S9.** Combined set of gene fusions in FFPE_Bx. **Table S11.** High confidence fusions detected in DKFZ_RP. **Table S12.** Overlap of fusions of all four cohorts. This table extents table 2 of the main manuscript with the DKFZ_RP cohort. **Figure S5.** Read coverage of exemplary snRNA::snoRNA gene fusions. **Figure S8.** Read coverage of potential novel gene fusions.

## Data Availability

All gene expression studies were obtained from the Gene Expression Omnibus (GEO) database. GEO series GSE134168 and GSE220095 (https://www.ncbi.nlm.nih.gov/geo/query/acc.cgi?acc=GSE220095) [Reviewer access token: gvyleqsavfavjil]) provide access to raw read counts of the cohorts FF_RP and FFPE_Bx, respectively. Raw read counts of the tumor-free samples of the FF_RP cohort are stored in GSE134073. Information on TCGA_PRAD can be found at http://cancergenome.nih.gov and https://www.cbioportal.org. The DKFZ_RP cohort is available at https://www.cbioportal.org and https://ega-archive.org (dataset ID: EGAD00001004791).

## Abbreviations

BCR: Biochemical recurrence
DB: Database
DoD: Death of diseaseD
DKFZ: Deutsches Krebsforschungszentrum
DKFZ_RP: Cohort; readical prostatectomy specimens from DKFZ
FFPE: Formalin-fixed paraffin-embedded
FFPE_Bx: Cohort; formalin-fixed paraffin-embedded biopsy specimens
FF_RP: Cohort; fresh-frozen radical prostatectomy specimens
GGG: Gleason grading group
GS: Gleason score
lncRNA: Long non-coding RNA
ncRNA: Non-coding RNA
PCa: Prostate cancer
PCR: Polymerase chain reaction
PSA: Prostate specific antigen
pN: Presence of regional lymph node metastases
RNA: Ribonucleic acid
snoRNA: Small nucleolar RNA
snRNA: Small nuclear RNA
TCC: Tumor cell content
TCGA: The Cancer Genome Atlas
TCGA_PRAD: Cohort; The Cancer Genome Atlas prostate adenocarcinoma tissue
