# Supplementary material for: Characterization and evaluation of gene fusions as a measure of genetic instability and disease prognosis in prostate cancer
Source: BMC Cancer. 2023 Jun 22;23:575. doi: 10.1186/s12885-023-11019-6 (PMC10286324; doi:10.1186/s12885-023-11019-6)
Supplement: Supplementary file 1 — Additional file 1: This document contains additional tables (Tables S1–S13) and figures (Figures S1–S11) to support the findings of our study. Tables and figures that are too extensive are provided as separate files; their captions, however, can be found here. Extra files provided: Table S3. Whitelist of known fusions as used with Arriba. Table S6. High confidence gene fusions of TCGA_PRAD and FF_RP. Table S7. Overlap between discovery cohorts TCGA_PRAD and FF_RP. Table S8. snoRNA gene fusions detected in all samples of FF_RP. Table S9. Combined set of gene fusions in FFPE_Bx. Table S11. High confidence fusions detected in DKFZ_RP. Table S12. Overlap of fusions of all four cohorts. This table extents table 2 of the main manuscript with the DKFZ_RP cohort. Figure S5. Read coverage of exemplary snRNA::snoRNA gene fusions. Figure S8. Read coverage of potential novel gene fusions. [file 12885_2023_11019_MOESM1_ESM.pdf]

Additional File 1

1 Supplementary Methods

1.1 Wet-lab confirmation of individual candidate gene fusions

To evaluate plausibility of detected snRNA::snoRNA fusions we chose exemplary fusions with highest supporting read counts for polymerase chain reaction (PCR). Primers were designed using the software Primer3 with the transcript sequences predicted by Arriba (Supplementary fig. S2).

The PCR was performed with two different master mixes:

Table 1 – Composition of PCR master mixes.

KAPA HiFi Master mix	Q5 Master mix
12.5µl Master mix	12.5µl Master mix
0.75µl Primer mix 10µM	1.25µl Primer mix 10µM
5µl template 1:10	5µl template 1:10
6.75µl H_2O	6.25µl H_2O

Table 2 – PCR runs for both master mixes.

KAPA-PCR		Q5-PCR	
95°C 3min		98°C 30s	
98°C 20s	35x	98°C 10s	35x
60°C 15s		55°C 15s	
72°C 15s		72°C 15s	
72°C 1min		72°C 2min	

The products of the PCR were loaded on a 2% agarose-gel in 0.5xTBE-buffer and run in an electrophoresis with 0.5xTBE as run-buffer for 1 hour at 150V. As ladders we used NEBs 100bp and 1kb ladders.

After electrophoresis, bands were cut and purified using QIAquick Gel Extraction Kit (qiagen). Finally, sanger sequencing was carried out at the core unit DNA technologies of the university of Leipzig.

2 Supplementary Tables

Table S1 – Composition of FF_RP. This table shows the inclusion criteria of the cohort FF_RP. The columns depict the internal risk group (column 1) based on value of the Gleason score (column 2) and involvement of the lymph nodes (column 3) with pN0 = no involvement and pN+ = lymph node involvement. Also, the survival status leading to inclusion in a certain risk group is shown (column 4). Column 5 lists the numbers of patients included per risk group with V+L+M+H_{tumor} = 40 tumor samples and C+$H_{tumor\text{-}free}$=24 control samples resulting in 64 samples overall.

Risk group	Gleason score (GS)	Lymphnode (pN)	Survival	Number
Control (C)	-	-	alive	8
Very low (V)	GS < 7	-	alive	8
Low (L)	GS = 7	-	alive	8
Medium (M)	GS ≤ 7	pN+	alive and DoD	8
High (H)	GS > 7	pN0 and pN+	alive and DoD	16 pairs with tumor-free samples

Table S2 – Clinical summary of cohorts. Additional information on technical and clinicopathological characteristics of the cohorts FF_RP (column 2), FFPE_Bx (column 3), TCGA_PRAD (column 4) and DKFZ_RP (column 5).

	FF_RP (n=40)	FFPE_Bx (n=176)	TCGA_PRAD (n=332)	DKFZ_RP (n=82)
Follow-up time, years median (1st and 3rd quartile)	12.2 (10.3, 15.3)	9.1 (5.7, 10.0)	1.5 (0.7, 2.8)	3.1 (2.1, 4.1)
Age in years				
<= 50	0	2	21	76
51-59	9	32	114	6
60-69	25	94	166	0
>=70	6	51	31	0
Presurgical total PSA in ng/ml median (1st and 3rd quartile)	10.8 (6.9, 18.4)	8.2 (5.6, 13.9)	7.6 (5.2, 12.1)	8.3 (6.2, 19.3)
Specimen Age in years (1st and 3rd quartile)	NA	9.4 (7.2, 10.1)	NA	NA
Gleason grade group				
<3	22	118	122	61
>=3	18	58	210	21
Pathological stage				
pT1/pT2	21	117	111	56
pT3/ pT4	19	59	214	26
NA	0	0	7	0
Lymph nodes				
tumor-negative	24	146	256	NA
tumor-positive	16	22	24	NA
NA	0	8	52	82
Tumor cell content median (1st and 3rd quartile)	77.5 (71.9, 83.1)	40 (20, 60)	70 (60, 80)	NA
Event				
BCR	NA	75	42	18
DoD	12	NA	4	NA
Sequencing depth (sequencing method)	~200 mio reads/sample (total RNA-seq)	~50 mio reads/sample (total RNA-seq)	~ 60 mio reads/sample (Poly(A)-seq)	NA (Poly(A)-seq)

Table S3 – Whitelist of known fusions as used with Arriba. This list is a combination of the list of known fusions as provided by the Arriba software and gene fusions described by Tandefelt et al. [1].

See text file: TS3_knownlist_ETSgenes.csv

Table S4 – Contingency table of fusions as detected by Arriba and published by the TCGA consortium [2]. The columns in each table show the numbers of (A) *ERG*, (B) *ETV1*, (C) *ETV4* and (D) *FLI1* fusions for 224 samples, for which clinical data is available from the Cell, 2015 TCGA PRAD dataset at cBioportal [3] and that were included in the ProstaTrend analyses by Kreuz et al. [4]. The numbers depict the high confidence fusions and in parentheses the high and medium confidence fusions as annotated by Arriba.

A ERG, high confidence (high and medium confidence)

		TCGA consortium			
		ERG-fusion	No ERG-fusion	Sum	Percentage of high and (high+medium) confidence ERG-fusions
	ERG-fusion	80 (103)	0	80 (103)	35.7% (46.0%)
	No ERG-fusion	27 (4)	117 (117)	144 (121)	
Arriba	Sum	107 (107)	117 (117)		
	Percentage of ERG-fusions	47.8%			

B ETV1, high confidence (high and medium confidence)

		TCGA consortium			
		ETV1-fusion	No ETV1-fusion	Sum	Percentage of high and (high+medium) confidence ETV1-fusions
	ETV1-fusion	10 (10)	0 (0)	10 (10)	4.5% (4.5%)
	No ETV1-fusion	1 (1)	213 (213)	214 (214)	
Arriba	Sum	11 (11)	213 (213)		
	Percentage of ETV1-fusions	4.9%			

Table S4 – continued

C ETV4, high confidence (high and medium confidence)

		TCGA consortium			
		ETV4-fusion	No ETV4-fusion	Sum	Percentage of high and (high+medium) confidence ETV4-fusions
**Arriba**	ETV4-fusion	6 (7)	1 (1)	7 (8)	3.1% (3.6%)
	No ETV4-fusion	1 (0)	216 (216)	217 (216)	
	Sum	7	217		
	Percentage of ETV4-fusions	3.1%			

D FLI1, high confidence (high and medium confidence)

		TCGA consortium			
		FLI1-fusion	No FLI1-fusion	Sum	Percentage of high and (high+medium) confidence FLI1-fusions
**Arriba**	FLI1-fusion	0 (0)	0 (0)	0 (0)	0% (0%)
	No FLI1-fusion	2 (2)	222 (222)	224 (224)	
	Sum	2 (2)	222 (222)		
	Percentage of FLI1-fusions	0.9%			

Table S5 – Comparison of gene fusions with specific known genes. Percentages of fusions with the genes ERG, ETV1, ETV4 and FLI1 as published by the TCGA consortium [2] (column 1) and as detected by Arriba with high confidence in our study in the three cohorts TCGA_PRAD (column 2), FF_RP (column 3) and FFPE_Bx (column 4). The results for the early onset PCa cohort DKFZ_RP are shown in column 5.

	TCGA_PRAD consortium	TCGA_PRAD Arriba	FF_RP	FFPE_Bx	DKFZ_RP
ERG	47.8%	35.7%	50%	14.8%	58.5%
ETV1	4.9%	4.5%	7.5%	2.3%	4.9%
ETV4	3.1%	3.1%	2.5%	0%	2.4%
FLI1	0.9%	0%	0%	0%	0%

Table S6 – High confidence gene fusions of TCGA_PRAD and FF_RP. Output of Arriba (merged <sample>.fusions.tsv files) for the discovery cohorts TCGA_PRAD and FF_RP, filtered by column **confidence** = high. In addition to standard Arriba columns (description see https://arriba.readthedocs.io/en/latest/output-files/ [5]), we added the column **source** denoting the cohort, the column **Fusion** combining the columns of **X.gene1** and **gene2** , the column **mitelman**, supplying information whether or not the fusion can be found in Mitelman DB, as well as the column **snoRNA**, that provides information, whether the involved genes are snoRNAs or snoRNA host genes.

See file: TS6_Discovery_fusions.csv

Table S7 – Overlap between discovery cohorts TCGA_PRAD and FF_RP. Output of Arriba (merged <sample>.fusions.tsv files) for the cohorts TCGA_PRAD and FF_RP. Filtered by column **confidence** = high and showing only fusions detected in both discovery cohorts. In addition to standard Arriba columns (description see https://arriba.readthedocs.io/en/latest/output-files/ [5]), we added the **source** column that describes the cohort, the **Fusion** column, combining **X.gene1** and **gene2** columns, the column **mitelman**, supplying information whether or not the fusion can be found in Mitelman DB, as well as the column **snoRNA**, that provides information on the involved genes in regard to being snoRNAs or snoRNA hosts.

See file: TS7_Discovery_fusions_overlap.csv

Table S8 – snoRNA gene fusions detected in all samples of FF_RP. Output of Arriba (merged <sample>.fusions.tsv files) of the cohort FF_RP. The table includes snoRNA or snoRNA hostgene gene fusions of all confidence levels of FF_RP. In addition to standard Arriba columns (description see https://arriba.readthedocs.io/en/latest/output-files/ [5]), we added the **Sample** column with the original Sample ID, the **Fusion** column, combining **X.gene1** and **gene2** columns, the column **snoDB_status**, that provides information on the involved genes in regard to being snoRNAs or snoRNA hosts, as well as the column **Group** carrying sample-type information (control, tumor-free or tumor).

See file: TS8_FF_RP_snoRNAfusions_allconf.csv

Table S9 – Combined set of gene fusions in FFPE_Bx. Output of Arriba (merged <sample>.fusions.tsv files) of the cohort FFPE_Bx. The table includes high confidence gene fusions of FFPE_Bx (column **confidence** = high, column **source** = High) as well as those fusions from the discovery cohorts fusionset, that could be detected in FFPE_Bx in all confidence levels (column **source** = Rec). In addition to standard Arriba columns (description see https://arriba.readthedocs.io/en/latest/output-files/ [5]), we added the **source** column (description: see above), the **Fusion** column, combining **X.gene1** and **gene2** columns, the column **mitelman**, supplying information whether or not the fusion can be found in Mitelman DB, as well as the column **snoRNA**, that provides information on the involved genes in regard to being snoRNAs or snoRNA hosts.

See file: TS9_Recall_fusions.csv

Table S10 – Multivariate Cox regression with dichotomized fusion numbers as well Gleason Grading and revised ProstaTrend score. As an additional analysis, we caluclated Cox regression including a third variable: a prognostic gene score for PCa. As prognostic score we utilized the revised ProstaTrend (PT) score [4]. It is an RNA-expression-based score to estimate prognosis for aggressive prostate cancer. In its revised version, only genes independent from specimen age in the FFPE biopsy cohort (FFPE_Bx) are included in the score. Columns represent the results per cohort, with all included TCGA_PRAD samples (n=332, column 1) and the combined FFPE_Bx dataset (n=176, column 2) as well as the FF_RP tumor samples (n=40, column 3),. The rows represent the tested variables with logHR and 95% confidence interval (CI), and p-value, respectively. For the variable of total fusions, the median per dataset is also recorded in a row. logHR ... logarithmic hazard ratio, CI ... confidence interval, p ... p-value.

		TCGA_PRAD (n=332, e=42)	FFPE_Bx (n=176, e=75)	FF_RP (n=40, e=12)
total fusions	median	7	2	12
	logHR (95% CI)	0.68 (-0.02, 1.38)	0.55 (0.09, 1.01)	-0.9 (-2.14, 0.34)
	p	0.05798	0.0193	0.15566
GGG	logHR (95% CI)	0.47 (0.13, 0.81)	0.61 (0.39, 0.83)	0.39 (-0.19, 0.97)
	p	0.00656	3.25e-08	0.18995
Revised PT	logHR (95% CI)	0.96 (-0.03, 1.96)	0.45 (-0.4, 1.3)	4.18 (1.51, 6.85)
	p	0.05803	0.2968	0.00216

Table S11 – High confidence fusions detected in DKFZ_RP. This table shows the frequency **n** of detected high confidence gene fusions in DKFZ_RP, the proportion of affected samples (**Percentage**) and whether the fusion can be found in the Mitelman database (**mitelman**).

See file: TS11_DKFZ_fusionfrequency.csv

Table S12 – Overlap of fusions of all four cohorts. This table extents table 2 of the main manuscript with the DKFZ_RP cohort.

See file: TS12_Overlap_4cohorts.csv

Table S13 – Overview of the analysis results for DKFZ_RP. In table (A) an overview over the numbers of detected fusions in each confidence level is given. Table (B) lists the results of the calculation of a Cox regression with dichotomized fusion numbers and continuous Gleason Grading. With table (C) we checked the consistency of *TMRPSS2::ERG* fusions among the samples of DKFZ_RP with replicates. The fusion is consistent among all replicates, it was found in either all or none of the replicates.

A

		**DKFZ_RP tumor**
	number of samples	82
all confidences	number of fusions	2,168
	average fusions per sample	26.44
	number of unique fusions per cohort	1,892
high	number of fusions	716 (33.03%)
	average fusions per sample	8.73
	number of unique fusions per cohort	666
medium	number of fusions	420 (19.37%)
	average fusions per sample	5.12
	number of unique fusions per cohort	357
low	number of fusions	1,158 (53.41%)
	average fusions per sample	14.12
	number of unique fusions per cohort	965

B

		DKFZ_RP (n=82, e=18)
total fusions	median	6
	logHR (95% CI)	1.45 (0.52, 4.07)
	p	0.482
GGG	logHR (95% CI)	1.99 (1.41, 2.81)
	p	0.000104

Table S13 – continued

C

Patient	Replicates	TMPRSS2::ERG status
ICGC_PCA034	6	0
ICGC_PCA035	6	6
ICGC_PCA038	6	6
ICGC_PCA041	5	0
ICGC_PCA044	3	0
ICGC_PCA037	2	2

3 Supplementary Figures

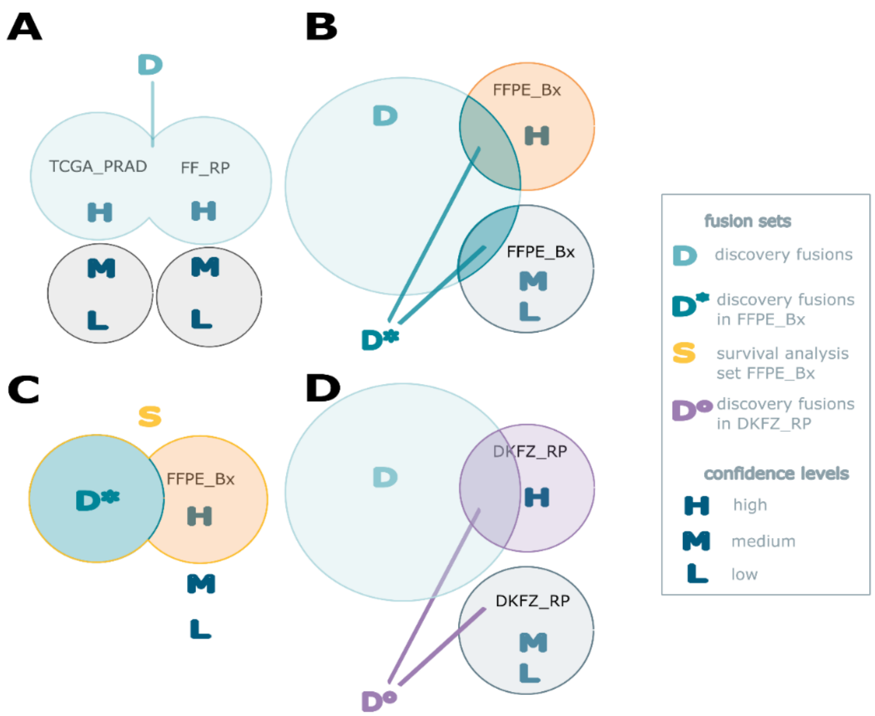

Figure S1 – Overview of the composition of fusions sets for the different cohorts and analyses. (A) All high confidence gene fusions of the cohorts TCGA_PRAD and FF_RP define the discovery fusion set "D". (B) Definition of discovery cohort fusion set in FFPE_Bx "D*": "D*" comprises all fusions of the set "D" that can be found in FFPE_Bx as high confidence as well as medium and low confidence fusions. (C) The set of fusions of FFPE_Bx used for survival analyses "S" consisting of "D*" fusions and all other FFPE_Bx high confidence fusions. (D) shows the definition of the discovery cohort fusion set in DKFZ_RP "D°": "D°" is comprised of all fusions of the set "D", that can be found in DKFZ_RP with high confidence.

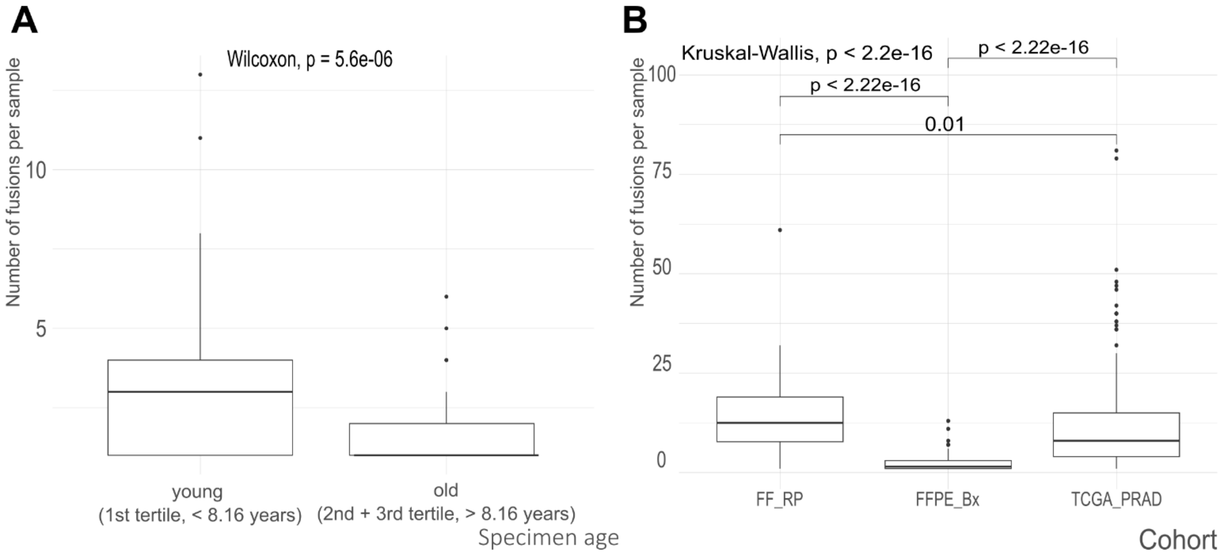

Figure S2 – Boxplots of fusion numbers. (A) This Boxplot shows the numbers of fusions detected per sample in FFPE_Bx divided by sample age into young (lowest tertile) and older (upper two tertiles) specimens. Plot (B) depicts the detected fusions per sample in each cohort. Pairwise tests are Wilcoxon tests.

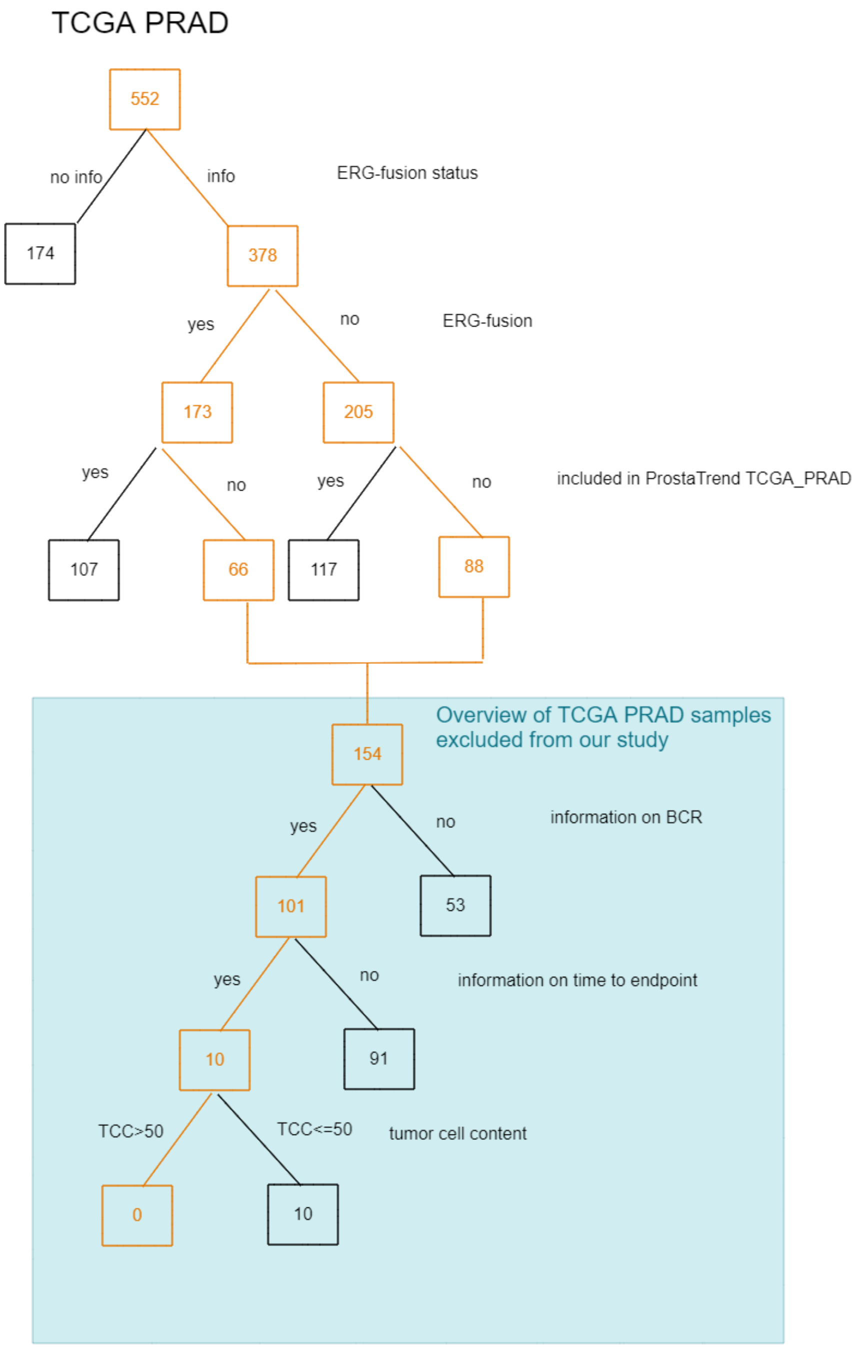

Figure S3 – Overview of inclusion criteria of samples from the TCGA_PRAD cohort. 154 samples did not meet inclusion criteria w.r.t. clinical follow-up and tumor cell content and where thus excluded from our study.

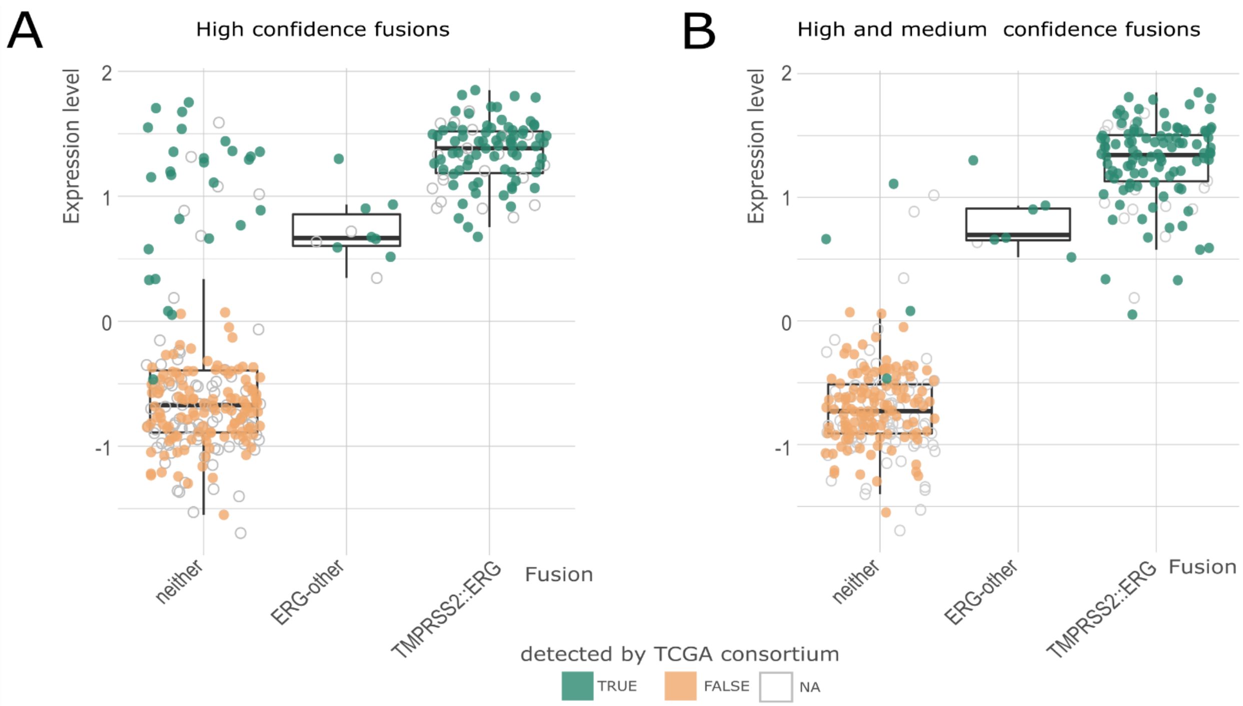

Figure S4 – ERG expression in TCGA_PRAD with and without fusion. It is known that expression level of *ERG* increases with *TMPRSS2* gene fusion [6]. We confirmed this increase in our TCGA_PRAD expression data paired with our fusion results. We assessed gene expression as described in the supplement of Kreuz et al. [4] and created a dot- and boxplot with the fusion status of *ERG* on the x-axis and quantified *ERG* expression on the y-axis and colored the dots (samples) according to their fusion status as published by the TCGA consortium [2]. Box- and dotplots of *ERG* expression sorted by fusion into three groups (as detected by Arriba): (1) *TMPRSS2::ERG* fusion (label: TMPRSS2::ERG), (2) fusion of *ERG* with another gene than TMPRSS2 (label: ERG-other) and (3) no such fusion (label: neither). Two levels of fusion confidence were examined, high confidence fusions in (A) and medium and high confidence fusions in (B). The coloring in both plots shows the published *ERG*-fusions of the TCGA consortium (green = TRUE, detected as fusion by TCGA; orange = FALSE, not detected as fusion by TCGA) as reported cBioportal [3]. For 23 of 27 samples for which Arriba did not report a high confidence *ERG*-fusion but high *ERG* expression (A) we detected a medium confidence fusion of *ERG* by our pipeline (B). Of the 4 samples for which we could not detect a fusion with Arriba, two samples exhibited high expression, the other two low *ERG* expression (B). DB ... database

See PDF: FS5_RNUSNO_reads.pdf

Figure S5 – Read coverage of exemplary snRNA::snoRNA gene fusions. The fusion calling software Arriba, used in this study, provided an R-script for the visualization of the detected fusions per sample. In this file the output of the Arriba visualization script is shown for two fusions, that were formed of snRNA and snoRNA. The results originate from one FF_RP sample. On top of each page, the location and breakpoint of a respective fusion are shown. Below that,

read coverage, orientation of the fusion partners and involved exons, as well as combined exons and sequence at the breakpoint can be found. In the bottom left corner, a Circos [7] plot shows the location of the fusion partners in the genome and on the bottom right the numbers of supporting reads are listed. Split reads: reads that map to each of the involved genes, the predominant gene is listed. Discordant mates: read pair, where one read maps to one partner and the second read to the other.

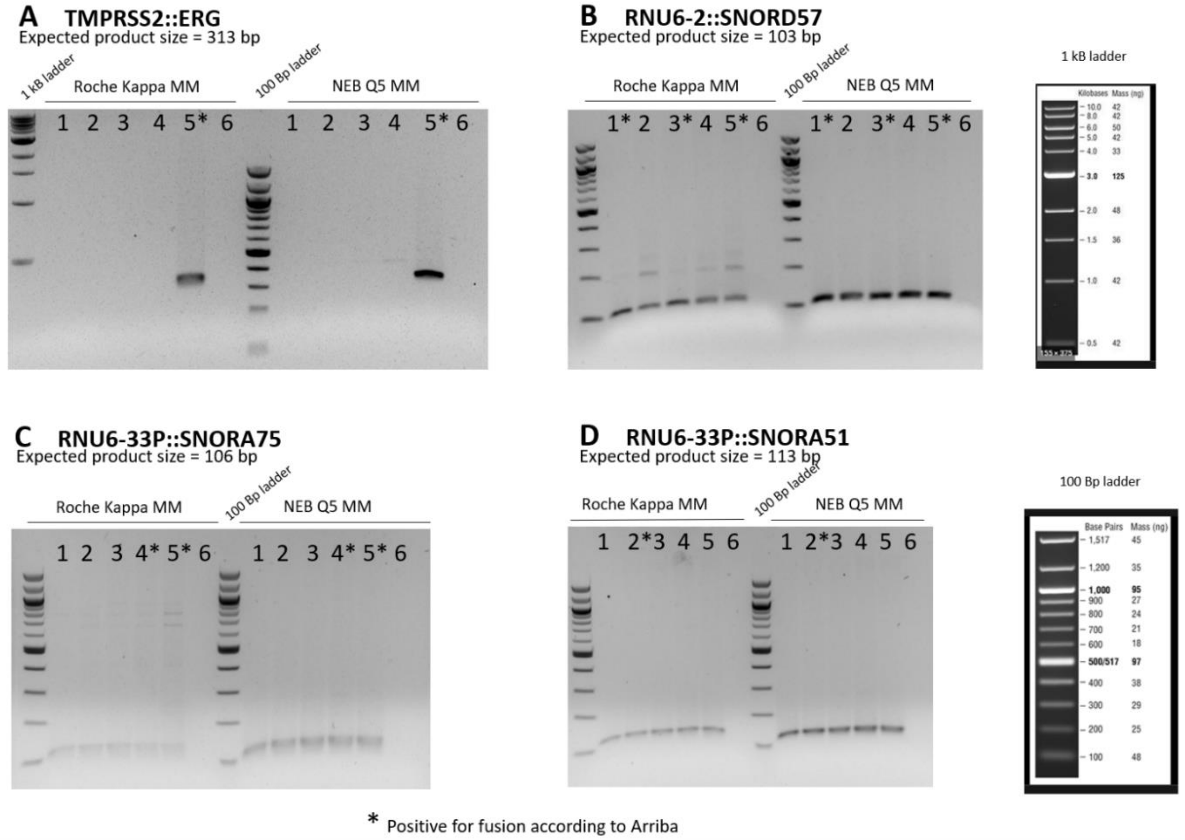

Figure S6 – In vitro verification of selected non-coding RNA gene fusions. Lane numbers correspond to the same samples. Lane 6 was an empty control. (A) Positive control with in silico *TMPRSS2::ERG* positive sample 5 and 4 negative samples. (B) Gel with the PCR results for *RNU6-2::SNORD57* (C) Results for *RNU6-33P::SNORA75* and (D) Results for *RNU6-33P::SNORA51*. The original, uncropped gel pictures can be found in additional files FS6a_original.png, FS6b_original.png, FS6c_original.png, and FS6d_original.png for the corresponding panels. Bp… base pairs, kB… kilobase, MM… Mastermix

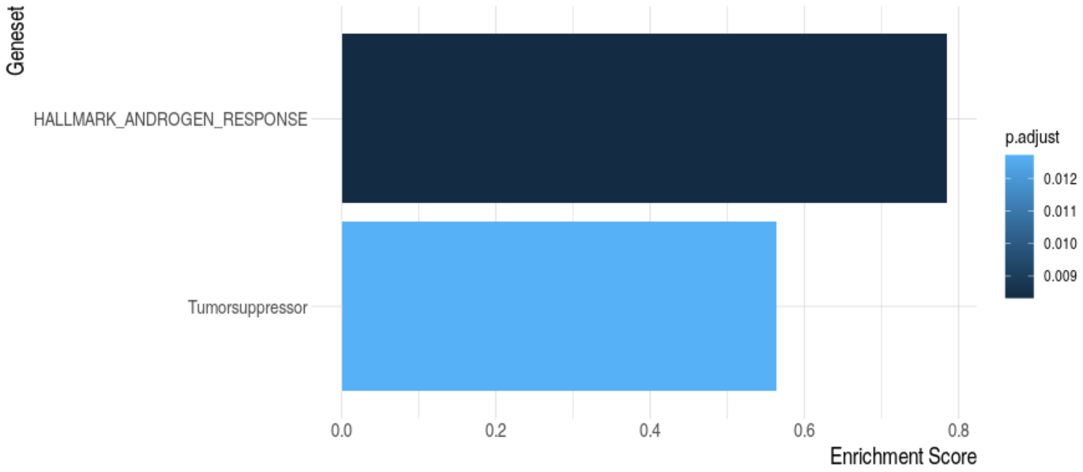

Figure S7 – Significant gene sets of Gene set enrichment analysis (GSEA) of fusions detected in the discovery cohorts. GSEA was performed with clusterProfiler (function GSEA()). Gene weights were determined by frequency in the fusion set, with singular hits per sample. Hallmark set Androgen response: p = 0.008 (adjusted with Benjamini-Hochberg procedure). Tumorsuppressor set: p = 0.013 (adjusted with Benjamini-Hochberg procedure). The analyzed gene sets were obtained from MsigDB R-package (filtered to HALLMARK sets) and TSGene DB for tumor suppressor genes [8, 9].

See PDF: FS8_Fusion_reads4.pdf

Figure S8 – Read coverage of potential novel gene fusions. The fusion calling software Arriba, used in this study, provides an R-script for the visualization of the detected fusions per sample. The file shows the Arriba visualization script output of selected FF_RP samples that carry the potential novel gene fusions detected in all cohorts. On top of each page, the location and breakpoint of a respective fusions are shown. Below that, read coverage, orientation of the fusion partners and involved exons, as well as combined exons and sequence at the breakpoint can be found. In the bottom left corner, a Circos [7] plot shows the location of the fusion partners in the genome and on the bottom right the numbers of supporting reads are listed. Split reads: reads that map to each of the involved genes, the predominant gene is listed. Discordant mates: read pair, where one read maps to one partner and the second read to the other.

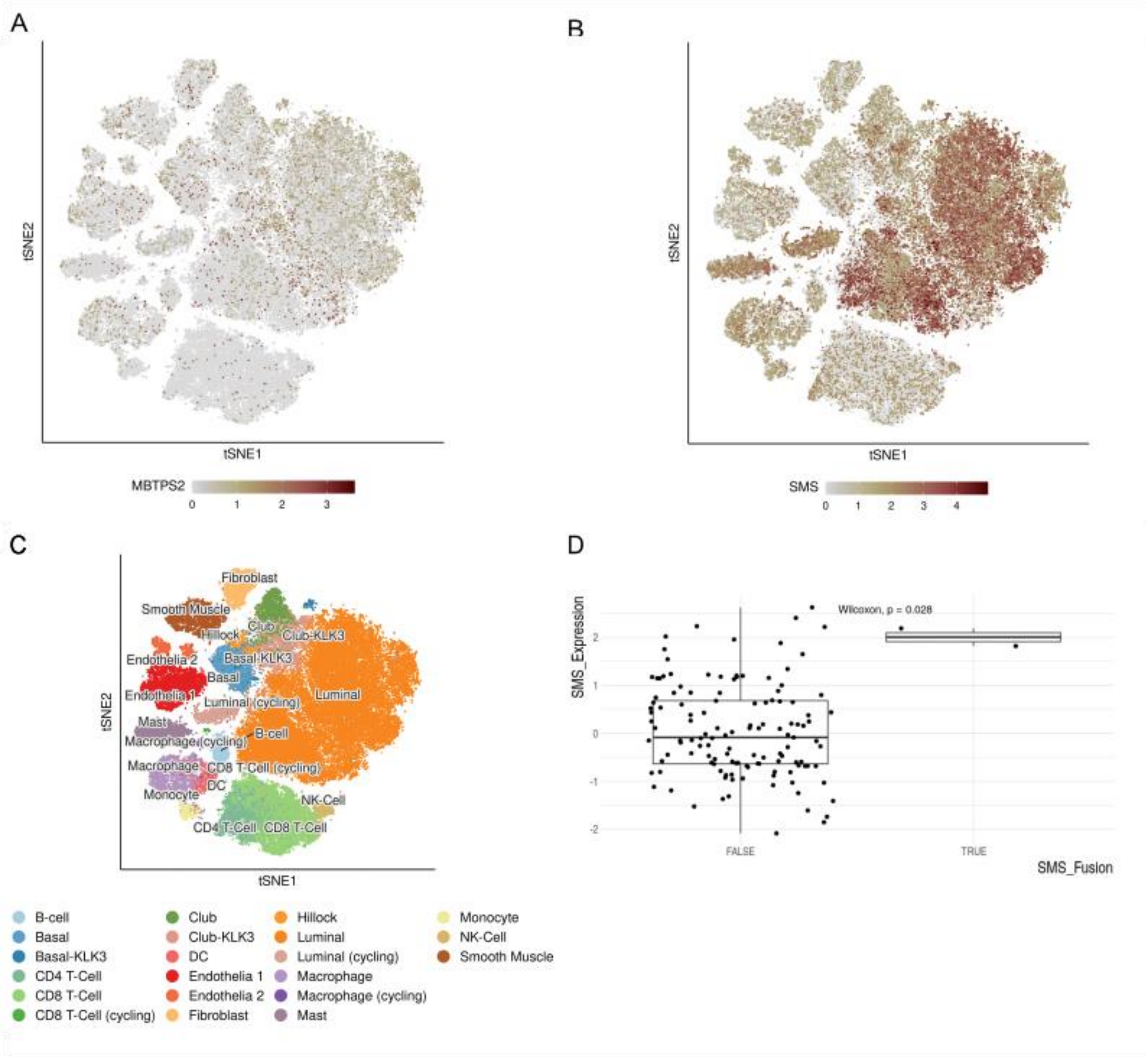

Figure S9 – Expression of genes involved in fusion based on single-cell sequencing data of the PCa cell Atlas [10]. The PCa cell atlas (https://bioinf.izi.fraunhofer.de/prostatrend/) contains single-cell sequencing data of studies by Chen et al. (GSE141445), Dong et al. (GSE137829), Ma et al. (GSE157703), Song et al. (GSE176031) and Tuong et al. (https://www.prostatecellatlas.org) [11–15]. The gene expression is study-wise standardized and normalized. The detailed methods are described by Rade et al. [10](A) Expression of the gene MBTPS2, (B) expression of the gene SMS, as shown in the ProstaTrend Atlas derived from the cohorts. (C) shows the distribution of annotated cell types. The cell types are annotated via a correlation-based approach, expression matched with specific cell type markers as well as published annotations. A more in-depth description can be found in the Additional File 1 of Rade et al. [10](page 26). Plot (D) shows the vst-standardized SMS expression and fusion status of TCGA_PRAD.

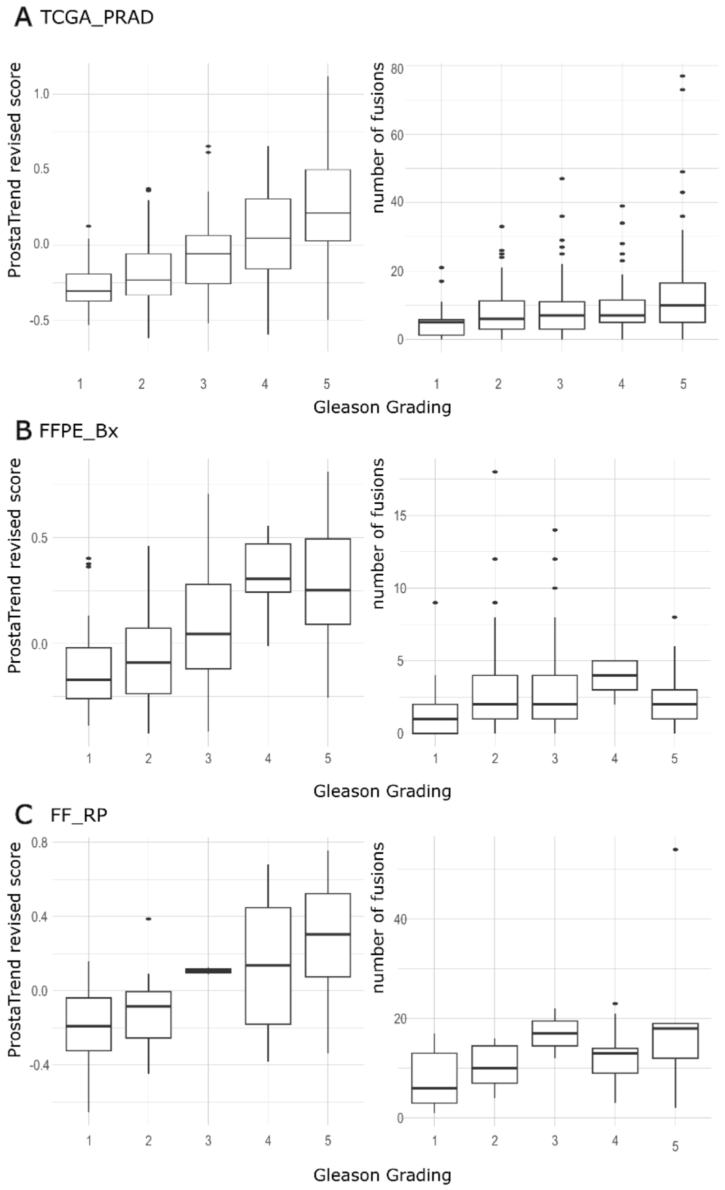

Figure S10 – Correlation of variables. We plotted boxplots with Gleason Grading groups versus ProstaTrend revised score and number of fusions per sample, respectively. With these plots we investigated the correlation between the variables to achieve further insights in the Cox regression results, as in Cox regression, when two variables strongly correlate, significance can fluctuate between the variables. The boxplots show strong correlation for Gleason Grading groups and ProstaTrend revised score indicating a strong association of adverse morphology and adverse transcriptomic signals. In contrast, no clear correlation between Gleason Grading groups and number of gene fusions is observed. (A) TCGA_PRAD, (B) FFPE_Bx and (C) FF_RP.

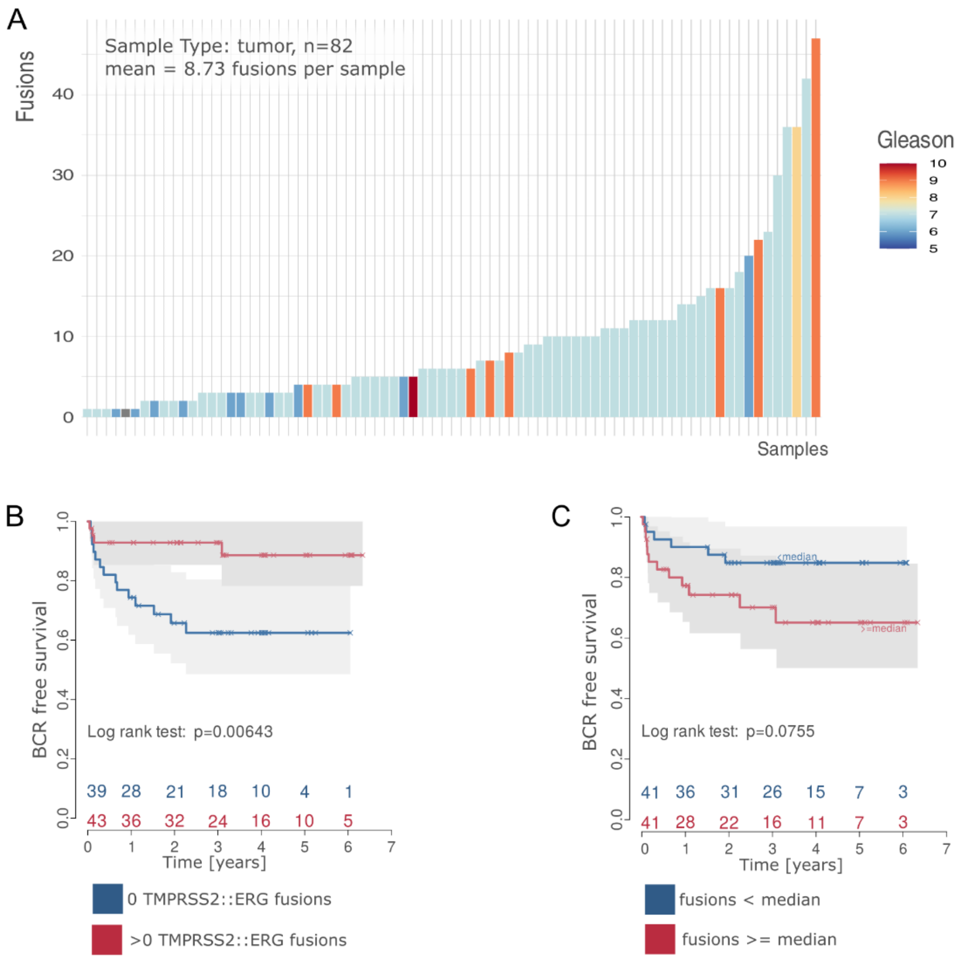

Figure S11 Overview of the analysis results for the DKFZ_RP cohort. Plot (A) shows the individual samples (n=82) ordered by the number of high confidence gene fusions. The coloring indicates the Gleason score of each sample. Panel (B) shows Kaplan-Meier curves and log-rank test for BCR-free survival for *TMPRSS2::ERG*-positive (red) and -negative (blue) patients. Plot (C) depicts the survival analyses with regard to the overall number of gene fusions above or equal to (red) and below median (blue).

4 References

1. Gasi Tandefelt D, Boormans J, Hermans K, Trapman J. ETS fusion genes in prostate cancer. Endocr. Relat. Cancer. 2014;21:R143-52.
2. Abeshouse A, Ahn J, Akbani R, Ally A, Amin S, Andry CD, et al. The Molecular Taxonomy of Primary Prostate Cancer. Cell. 2015;163:1011–25.
3. Gao J, Mazor T, Ciftci E, Raman P, Lukasse P, Bahceci I, et al. Abstract 923: The cBioPortal for Cancer Genomics: An intuitive open-source platform for exploration, analysis and visualization of cancer genomics data. Cancer Res. 2018;78:923.

4. Kreuz M, Otto DJ, Fuessel S, Blumert C, Bertram C, Bartsch S, et al. ProstaTrend-A Multivariable Prognostic RNA Expression Score for Aggressive Prostate Cancer. Eur. Urol. 2020;78:452–9.
5. Arriba Documentation Outputfiles. https://arriba.readthedocs.io/en/latest/output-files/.
6. Song C, Chen H. Predictive significance of TMRPSS2-ERG fusion in prostate cancer: a meta-analysis. Cancer Cell Int. 2018;18:177.
7. Gu Z, Gu L, Eils R, Schlesner M, Brors B. circlize Implements and enhances circular visualization in R. Bioinformatics. 2014;30:2811–2.
8. Liberzon A, Birger C, Thorvaldsdóttir H, Ghandi M, Mesirov JP, Tamayo P. The Molecular Signatures Database (MSigDB) hallmark gene set collection. Cell Syst. 2015;1:417–25.
9. Zhao M, Kim P, Mitra R, Zhao J, Zhao Z. TSGene 2.0: an updated literature-based knowledgebase for tumor suppressor genes. Nucleic Acids Res. 2016;44:D1023-31.
10. Michael Rade, Markus Kreuz, Angelika Borkowetz, Ulrich Sommer, Conny Blumert, Susanne Fuessel, Catharina Bertram, Dennis Löffler, Dominik J. Otto, Livia A. Wöller, Carolin Schimmelpfennig, Ulrike Köhl, Ann-Cathrin Gottschling, Pia Hönscheid, Gustavo B. Baretton, Manfred Wirth, Christian Thomas, Friedemann Horn, Kristin Reiche. The prognostic gene-expression signature ProstaTrend adapted to Formalin-Fixed Paraffin-Embedded biopsies of prostate cancer. In submission.
11. Chen S, Zhu G, Yang Y, Wang F, Xiao Y-T, Zhang N, et al. Single-cell analysis reveals transcriptomic remodellings in distinct cell types that contribute to human prostate cancer progression. Nat. Cell Biol. 2021;23:87–98.
12. Ma X, Guo J, Liu K, Chen L, Liu D, Dong S, et al. Identification of a distinct luminal subgroup diagnosing and stratifying early stage prostate cancer by tissue-based single-cell RNA sequencing. Mol. Cancer. 2020;19:147.
13. Song H, Weinstein HNW, Allegakoen P, Wadsworth, 2nd, Marc H, Xie J, Yang H, et al. Single-cell analysis of human primary prostate cancer reveals the heterogeneity of tumor-associated epithelial cell states. Nat. Commun. 2022;13:141.
14. Dong B, Miao J, Wang Y, Luo W, Ji Z, Lai H, et al. Single-cell analysis supports a luminal-neuroendocrine transdifferentiation in human prostate cancer. Communications Biology. 2020;3:1–15.
15. Tuong ZK, Loudon KW, Berry B, Richoz N, Jones J, Tan X, et al. Resolving the immune landscape of human prostate at a single-cell level in health and cancer. Cell Rep. 2021;37:110132.
